# Tandem organic solar cells with 20.6% efficiency enabled by reduced voltage losses

**DOI:** 10.1093/nsr/nwad085

**Published:** 2023-03-30

**Authors:** Jianqiu Wang, Zhong Zheng, Pengqing Bi, Zhihao Chen, Yafei Wang, Xiaoyu Liu, Shaoqing Zhang, Xiaotao Hao, Maojie Zhang, Yongfang Li, Jianhui Hou

**Affiliations:** State Key Laboratory of Polymer Physics and Chemistry, Beijing National Laboratory for Molecular Sciences, Institute of Chemistry, Chinese Academy of Sciences, Beijing 100190, China; Laboratory of Advanced Optoelectronic Materials, Suzhou Key Laboratory of Novel Semiconductor-optoelectronics Materials and Devices, College of Chemistry, Chemical Engineering and Materials Science, Soochow University, Suzhou 215123, China; State Key Laboratory of Polymer Physics and Chemistry, Beijing National Laboratory for Molecular Sciences, Institute of Chemistry, Chinese Academy of Sciences, Beijing 100190, China; School of Chemistry and Biology Engineering, University of Science and Technology Beijing, Beijing 100083, China; State Key Laboratory of Polymer Physics and Chemistry, Beijing National Laboratory for Molecular Sciences, Institute of Chemistry, Chinese Academy of Sciences, Beijing 100190, China; State Key Laboratory of Polymer Physics and Chemistry, Beijing National Laboratory for Molecular Sciences, Institute of Chemistry, Chinese Academy of Sciences, Beijing 100190, China; School of Physics, State Key Laboratory of Crystal Materials, Shandong University, Jinan 250100, China; State Key Laboratory of Polymer Physics and Chemistry, Beijing National Laboratory for Molecular Sciences, Institute of Chemistry, Chinese Academy of Sciences, Beijing 100190, China; University of Chinese Academy of Sciences, Beijing 100049, China; State Key Laboratory of Polymer Physics and Chemistry, Beijing National Laboratory for Molecular Sciences, Institute of Chemistry, Chinese Academy of Sciences, Beijing 100190, China; School of Chemistry and Biology Engineering, University of Science and Technology Beijing, Beijing 100083, China; State Key Laboratory of Polymer Physics and Chemistry, Beijing National Laboratory for Molecular Sciences, Institute of Chemistry, Chinese Academy of Sciences, Beijing 100190, China; School of Chemistry and Biology Engineering, University of Science and Technology Beijing, Beijing 100083, China; School of Physics, State Key Laboratory of Crystal Materials, Shandong University, Jinan 250100, China; Laboratory of Advanced Optoelectronic Materials, Suzhou Key Laboratory of Novel Semiconductor-optoelectronics Materials and Devices, College of Chemistry, Chemical Engineering and Materials Science, Soochow University, Suzhou 215123, China; Laboratory of Advanced Optoelectronic Materials, Suzhou Key Laboratory of Novel Semiconductor-optoelectronics Materials and Devices, College of Chemistry, Chemical Engineering and Materials Science, Soochow University, Suzhou 215123, China; CAS Key Laboratory of Organic Solids, Beijing National Laboratory for Molecular Sciences, Institute of Chemistry, Chinese Academy of Sciences, Beijing 100190, China; University of Chinese Academy of Sciences, Beijing 100049, China; State Key Laboratory of Polymer Physics and Chemistry, Beijing National Laboratory for Molecular Sciences, Institute of Chemistry, Chinese Academy of Sciences, Beijing 100190, China; School of Chemistry and Biology Engineering, University of Science and Technology Beijing, Beijing 100083, China; University of Chinese Academy of Sciences, Beijing 100049, China

**Keywords:** organic solar cells, power conversion efficiency, voltage losses, tandem cells, ternary strategy

## Abstract

Large voltage losses are the main obstacle for achieving high efficiency in organic solar cells (OSCs). Here we construct ternary OSCs by introducing an asymmetric small molecule acceptor AITC into PBDB-TCl : BTP-eC9 system and demonstrate the effectiveness in simultaneously decreasing energy disorder and non-radiative voltage losses. It is found that the introduction of AITC can modify domain size and increase the degree of crystallinity, which enhances open-circuit voltage and power conversion efficiency (19.1%, certified as 18.9%). Inspiringly, an output efficiency of 20.6% of the constructed tandem OSCs based on PBDB-TCl : AITC : BTP-eC9 ternary active layer output a recorded efficiency of 20.6% (certified as 20.3%), which is the highest value in OSCs field to date. This work demonstrates that decreasing the voltage losses by ternary strategy and constructing of tandem architecture are effective approaches towards improving photovoltaic performance.

## Teaser

Tandem organic solar cells with recorded efficiency are achieved by implementing the ternary strategy to simultaneously reduce energy disorder and non-radiative voltage losses.

## INTRODUCTION

Organic solar cells (OSCs) have attracted wide attention because of their light weight, good mechanical flexibility and tunable semitransparency [1–4]. Power conversion efficiency (PCE) is a critical parameter for solar cell applications, and can be improved by material innovation and device engineering. Till now, the PCEs of single-junction and tandem OSCs have been improved to 19.6% and 20.2%, respectively [5,6]. However, there is still a gap of PCE between OSCs and other photovoltaic technologies due to the suppressive open-circuit voltage (*V*_OC_) imposed by the relatively large voltage losses (Δ*V*_loss_) [7–10]. To restrain the Δ*V*_loss_ is an important topic in the field of OSCs society.

Δ*V*_loss_ in a solar cell is defined as the difference between the optical bandgap divided by elemental charge (*E*_g_/*q*) and open-circuit voltage (*V*_OC_), which can be calculated quantitatively by the principle of detailed balance. As all these parameters are measurable, it is easy to compare the Δ*V*_loss_ in OSCs with other solar cells [8,11–14]. In particular, the radiative and non-radiative charge recombination constitutes major parts of the Δ*V*_loss_. The radiative voltage loss generally originates from two parts. The first is the radiative recombination above the optical gap (Δ*V*_1_), which is unavoidable for all solar cells [11]. The other radiative loss is ascribed to radiative recombination below the optical gap (Δ*V*_2_). This part can be suppressed by reducing the degree of energetic disorder or reorganization energy [8,14–16], and Δ*V*_2_ is already below 50 mV in the high-performance OSCs [8,14,17]. As another component of Δ*V*_loss_, the non-radiative recombination voltage loss (Δ*V*_3_) is intrinsically linked to the external electroluminescence quantum efficiency (EQE_EL_) of the OSCs [12,18,19]. In state-of-the-art OSCs, EQE_EL_ is roughly 10^−4^–10^−5^, corresponding to a Δ*V*_3_ of 0.23–0.29 V [20]. If over 21% and 24% PCE need to be achieved in single-junction and tandem OSCs, Δ*V*_3_ should be restrained to 0.15 V without sacrificing Δ*V*_2_.

Currently, innovation of materials and upgrading of device structures are the main strategies employed to reduce the Δ*V*_loss_ in OSCs [10,13,14,19]. For instance, the design and synthesis of ITIC derivatives boost the EQE_EL_ to the order of ∼10^−4^, which is much higher than that of fullerene-based OSCs (EQE_EL_ below 10^−6^). This leads to the reduction of Δ*V*_3_ and Δ*V*_loss_ to 0.40 V and 0.70 V, respectively, and PCE of ∼15% is obtained [21]. Recently, Y6-families non-fullerene materials show their EQE_EL_ reaching the order of 10^−4^, which gives rise to the Δ*V*_3_ and Δ*V*_loss_ of OSCs to 0.25 V and 0.55 V, respectively, and the corresponding PCEs are pushed to 18% [22,23]. In terms of device structure upgrades, the ternary strategy of introducing a third component into the binary active layers has been demonstrated as an efficient and convenient method to address the voltage loss [13,14,24]. On the other hand, tandem architecture emerges as a promising strategy to suppress Δ*V*_3_ and Δ*V*_loss_ that originated from the vibrational relaxation of hot excitons in single-junction OSCs [25,26]. In practice, although there have been valuable simulation and experimental studies on the effect of device optimization on Δ*V*_loss_ of OSCs, there is a lack of research focusing on reducing the Δ*V*_3_ without sacrificing the external quantum efficiency (EQE) edge; decreasing the Δ*V*_2_ by reducing the energy disorder and optimizing the morphology of the ternary active layer.

Here, we reduce the voltage losses and improve the PCE of OSCs by introducing an asymmetric small molecule acceptor (SMA) AITC as the third component into high-performance PBDB-TCl : BTP-eC9 binary blends. As expected, the addition of AITC reinforces the molecular packing and tunes the domain size. Consequently, we suppress charge recombination, expedite hole transfer and narrow down energetic disorder and electronic density of states in the active layer. Due to improvements in comprehensive performance, the ternary OSCs exhibit a high fill factor (FF) of 80.5% and a high *V*_OC_ of 0.88 V with significantly reduced Δ*V*_3_, an enormously boosted PCE of 19.1% (certified as 18.9%) is achieved. In addition, the ternary blend is used as the sub-cell active layer in tandem cells and outputs a recorded PCE of 20.6% (certified as 20.3%), which is the highest value for OSCs to date.

## RESULTS AND DISCUSSION

### Materials and device performance

**Figure 1.**
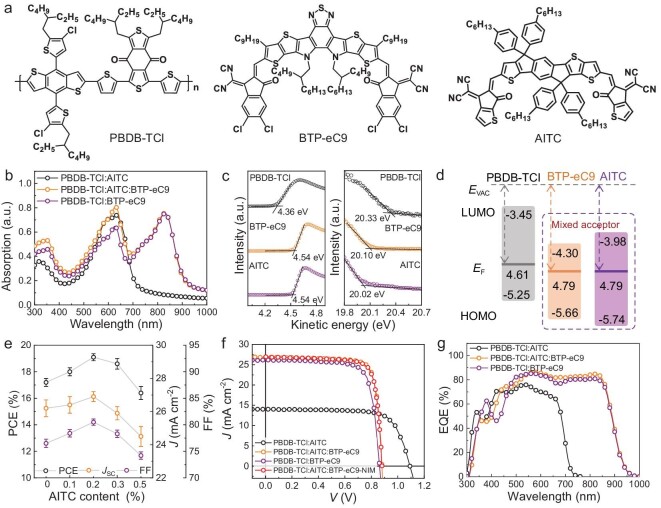
Materials characteristics and device photovoltaic performance. (a) Molecular structures of PBDB-TCl polymer donor and SMAs BTP-eC9 and AITC. (b) Absorption spectra of three neat films. (c) UPS results of PBDB-TCl (black line), BTP-eC9 (orange line) and AITC (purple line). (d) Energy level diagram and charge transport dynamic progress of PBDB-TCl, AITC and BTP-eC9. The solid lines and the numbers (4.61, 4.79, and 4.79 eV) represent Fermi energy level of PBDB-TCl, BTP-eC9 and AITC neat films. (e) PCE, *J*sc and FF values of OSCs based on PBDB-TCl : AICT : BTP-eC9 with different acceptor ratios (donor/acceptor = 1 : 1.2). Error bars represent the standard error of the mean (*n* = 10). (f) Current density*-*voltage (*J*-*V*) curves of binary and ternary OSCs. The certified result of optimal ternary OSC measured by NIM, China. (g) EQE curves of OSCs based on three blended films. The calculated current density (*J*_cal._) by integrating the EQE for the OSCs, which agrees well with that acquired from *J–V* measurements (about 1% errors).

The molecular structures of PBDB-TCl polymer donor, SMAs BTP-eC9 and AITC are shown in Fig. 1a. Figure 1b shows the absorption spectra of their binary and ternary blend films. The PBDB-TCl : AITC film exhibits strong absorption from 300 to 700 nm, which is well complementary with PBDB-TCl : BTP-eC9. The highest occupied molecular orbital (HOMO) and the lowest unoccupied molecular orbital (LUMO) energies of the three films are −5.25 eV and −3.45 eV for PBDB-TCl; −5.66 eV and −4.30 eV for AITC; and −5.74 eV and −3.98 eV for BTP-eC9, measured by ultraviolet photoelectron spectroscopy (UPS) (Fig. 1c–d). In our previous work, the asymmetric molecule AITC with large dipole moment shows good miscibility with BTP-eC9, which facilitates the formation of a stable mixing phase in blends [24]. Based on materials of three active layers, the single-junction OSC is fabricated. The photovoltaic parameters of OSCs based on the binary and ternary active layers with various ratios of components are provided in Fig. 1e–f, Table 1 and Fig. S1. PBDB-TCl : BTP-eC9–based binary OSCs exhibit a *V*_OC_ of 0.86 V, a short circuit current density (*J*_SC_) of 26.2 mA cm^−2^, an FF of 76.5% and a PCE of 17.2%. The PBDB-TCl : AITC-based binary OSCs show a *V*_OC_ of 1.09 V, *J*_SC_ of 14.0 mA cm^−2^, an FF of 72.6% and a PCE of 11.1%. Moreover, ternary OSCs with an active layer of optimal weight ratio (1 : 1.2 : 0.2) offers a *V*_OC_ of 0.88 V, a *J*_SC_ of 26.9 mA cm^−2^, an FF of 80.5% and a PCE of 19.1%. The PCE of the best ternary OSC is certified as 18.9% by NIM, China (Fig. S2, Fig. 1f and Table 1), which is the one of the highest certified results [6,23,27–30].

**Table 1.** Photovoltaic parameters of single-junction and tandem OSCs under the illumination AM 1.5 G, 100 mW cm^−2^.

OSCs	Active layer	Thickness (nm)	*V*_OC_ (V)	*J*_SC_/*J*_cal_.(mA cm^−2^)^a^	FF (%)	PCE (%)^b^
Single-junction	PBDB-TCl:AITC	117	1.09	14.0/13.9	72.6	11.1 (10.8 ± 0.3)
	PBDB-TCl:AITC:BTP-eC9	119	0.88	26.9/26.7	80.5	19.1 (18.7 ± 0.2)
	PBDB-TCl:AITC:BTP-eC9	119	0.88	26.9	79.6	18.9^c^
	PBDB-TCl:BTP-eC9	112	0.86	26.2/25.9	76.5	17.2 (16.8 ± 0.3)
Tandem	PFBCPZ:AITC/PBDB-TCl:AITC:BTP-eC9	80/130	2.03	12.5	77.6	19.7 (19.4 ± 0.2)
		90/130	2.02	13.3	76.6	20.6 (20.2 ± 0.3)
		90/130	2.02	13.2	76.0	20.3^b^
		100/130	2.02	12.7	74.8	19.2 (18.7 ± 0.4)
		90/120	2.03	12.8	77.1	20.0 (19.5 ± 0.3)
		90/140	2.01	13.7	73.7	20.2 (19.7 ± 0.2)
		80/130	2.03	12.5	77.6	19.7 (19.4 ± 0.2)
		90/130	2.02	13.3	76.6	20.6 (20.2 ± 0.3)

^a^Integrated current density based on EQE spectrum.

^b^Average values with standard deviation are obtained from 10 devices.

^c^Certified by National Institute of Metrology, China (NIM, China).

### Photophysical analysis

**Figure 2.**
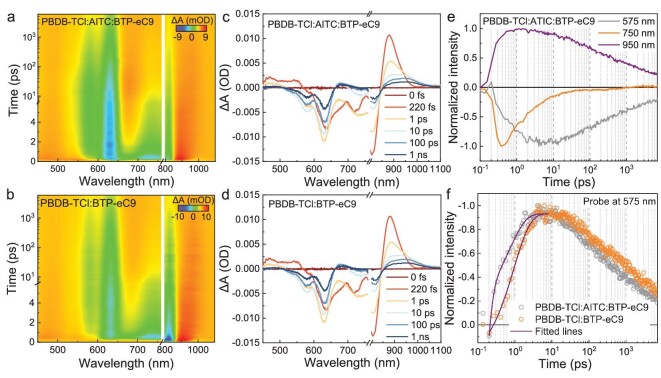
Photophysical characteristics and charge transport dynamics. (a and b) 2D TA spectra of PBDB-TCl : AITC : BTP-eC9 and PBDB-TCl : BTP-eC9 under 800 nm excitation. (c and d) TA spectra at different time delays of PBDB-TCl : AITC : BTP-eC9 and PBDB-TCl : BTP-eC9 under the excitation wavelength of 800 nm. (e) Decay dynamics probed at 575, 750 and 950 nm in PBDB-TCl : AITC : BTP-eC9 systems under 800 nm excitation. (f) Decay dynamics probed at 575 nm in PBDB-TCl : AITC : BTP-eC9 and PBDB-TCl : BTP-eC9 systems under 800 nm excitation.

Femtosecond transient absorption (TA) spectroscopy is utilized to investigate the effect of AITC on the charge transfer dynamics in the ternary active layers. The 660 and 800 nm pulses are used to excite neat AITC and BTP-eC9 films, respectively. As shown in Fig. S3, the main TA signals are observed at 500–700 nm and 600–900 nm for AITC and BTP-eC9 films, respectively. These peaks arise from the main optical transition from the ground-state bleaching (GSB) signals that progressively decrease with delay time [31,32]. We measure the TA profiles of blend films and the data are shown in Fig. 2a–d. Here, an 800 nm excitation pump pulse is used to solely excite the BTP-eC9 acceptor to obtain the hole transfer signals. The decay dynamics at various wavelengths represent the different photophysical processes, as shown in Fig. 2e–f and Fig. S4. The signals at 575 and 750 nm are assigned to the GSB signals of PBDB-TCl and BTP-eC9, respectively. The decay trace at 950 nm is the photoinduced absorption of singlet excitons of the acceptor. Figure 2e and Fig. S4a show that the bleaching signals at 575 nm appear in the TA spectra with signal decay at 750 nm, which confirms the hole transfer process from acceptor to donor. The decay dynamics at 575 nm are selected as the characteristic signals and fitted by using biexponential functions for comparing the rates of hole transfer in the blends. As shown in Fig. 2f and Table S2, the *τ*_1_ and *τ*_2_ values of ternary films are both lower than that of binary films, which indicate more rapid hole transfer processes [13,33]. These results demonstrate that the addition of AITC promotes the rate of hole transfer, which is beneficial to suppress the bimolecular recombination probability and improve photovoltaic performance of the corresponding OSCs.

### Analysis of device physics

**Figure 3.**
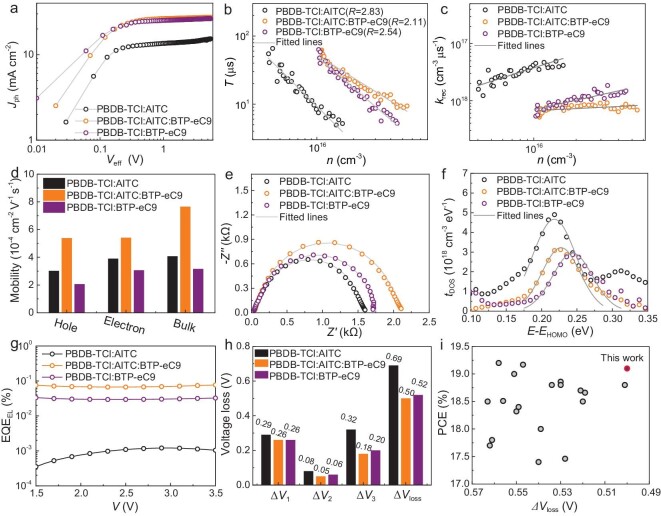
Device physics characterization. (a) Photocurrent (*J*_ph_) versus effective voltage (*V*_eff_) for three OSCs. (b and c) Charge lifetime (*τ*) and recombination rate coefficient (*R*_rec_) as a function of charge density (*n*) of the binary and ternary OSCs; *n* and *τ* are calculated by transient photovoltage (TPV) and charge extraction (CE) measurements. (d) Carrier mobility of blend films. The hole, electron and bulk carrier mobility are obtained by the space charge limited current (SCLC) method [40] and photo-induced charge-carrier extraction at linearly increasing voltage (photo-CELIV) measurement [41]. (e) Electrochemical impedance spectra for three devices. The fitted lines are obtained by fitting based on equivalent circuit model shown in Fig. S8, where *R*_s_, *R*_trans_ and *R*_rec_ represent the series resistances from all contacting interfaces, transport resistance and recombination resistance of active layer. (f) Defects density of state of the three films and corresponding fitting results. (g) EQE_EL_ curves of three OSCs. (h) Summarization of various voltage losses. (i) Plots of PCE against Δ*V*_loss_ (OSCs with PCEs > 17% including this work).

The exciton dissociation probability (*P*_diss_) is calculated according to the previously established method [34,35]. Figure 3a shows the photocurrent (*J*_ph_) as a function of the effective voltage (*V*_eff_) of three OSCs. Consequently, the *P*_diss_ of the three OSCs are 0.88, 0.96 and 0.93, respectively, which partially explains the improved FF in the ternary OSCs. In addition, to evaluate charge recombination kinetics, the charge carrier densities (*n*) and carrier lifetimes (*τ*) of the OSCs are calculated. As shown in Fig. S5 and Fig. 3b, the ternary devices exhibit higher *n* and *τ* along with lower recombination order (*λ*) compared to the binary OSCs, which indicates a mechanism close to an ideal bimolecular recombination, justified by the increased *J*_SC_ and FF in ternary OSCs [36–38]. Based on *n* and *τ*, the biomolecular recombination rate constant (*k*_rec_) can be calculated [37]. As shown in Fig. 3c, *k*_rec_ for the ternary device is still lower than that of the two binary devices, which indicates a significantly reduced bimolecular recombination loss, contributing to improved photoelectric properties of devices.

The charge transport properties are further evaluated, as shown in Fig. 3d, Figs. S6–7 and Table S3. The hole and electron mobility of the three films are 3.01 × 10^−4^ and 3.89 × 10^−4^ cm^2^ V^−1^ s^−1^ for PBDB-TCl : AITC, 5.37 × 10^−4^ and 5.40 × 10^−4^ cm^2^ V^−1^ s^−1^ for PBDB-TCl : AITC : BTP-eC9, 2.05 × 10^−4^ and 3.05 × 10^−4^ cm^2^ V^−1^ s^−1^ for PBDB-TCl : BTP-eC9, respectively. On the other hand, the ternary films also show higher bulk carrier mobility in photo-CELIV measurements (Table S3). The higher and more balanced mobility in ternary films is helpful in improving FF and *J*_SC_. Electrochemical impedance spectroscopy (EIS) measurements are also performed. As shown in Fig. 3e and Table S4, the ternary OSCs exhibit smaller *R*_s_ and *R*_trans_ as well as larger *R*_rec_, indicating promoted charge transport and restrained charge recombination, which is consistent with the improved FF and photovoltaic performance [33,39]. In addition, the electronic trap density of states (DoS) of OSCs are evaluated from the capacitance-frequency curves. As shown in Fig. 3f and Table S5, ternary OSCs show a smaller *σ* (0.027 eV) than that of binary OSCs (0.031 eV for PBDB-TCl : AITC and 0.028 eV for PBDB-TCl : BTP-eC9), corresponding to the narrower distribution, which indicates the suppressed energetic disorder. Moreover, the center of the DoS (*E*_t_) of PBDB-TCl : AITC, PBDB-TCl : AITC : BTP-eC9 and PBDB-TCl : BTP-eC9 are 0.22, 0.23 and 0.24 eV, respectively, which indicates that the addition of AITC gives rise to *E*_t_ shifts slightly to HOMO direction in the ternary OSCs. Consequently, a high *V*_OC_ of 0.88 V is obtained. These features can promote carrier transport since excitons and carriers trapped in the tail states of DoS are decreased, which can be ascribed to the growth of crystallites with better crystalline quality. The detailed morphology analysis will be discussed later.

### Analysis of voltage losses

To probe the enhanced *V*_OC_ in ternary OSCs, Δ*V*_loss_ of three OSCs are obtained by EQE_FTPS_ and EL spectra. The detailed processes are provided in Supporting Information (SI). The results of relevant characterizations and calculated parameters of Δ*V*_loss_ are summarized in Fig. 3 and Table S6. The optical band gaps of OSCs (*E*_gs, OSC_) are determined to be 1.78, 1.38 and 1.38 eV, respectively (Fig. S10). The introduction of AITC into PBDB-TCl : BTP-eC9 does not improve the *E*_g_. Moreover, ternary OSCs show a lower Δ*V*_loss_ of 0.50 V than that of the PBDB-TCl : AITC (0.69 V) and PBDB-TCl : BTP-eC9 (0.52 V) binary OSCs. Specifically, the unavoidable Δ*V*_1_ of the three devices are calculated to be 0.29, 0.26 and 0.26 V, respectively. Moreover, the ternary devices show a very low Δ*V*_2_ of 0.05 V, which can be attributed to the sharp band tail absorption dominated by low energetic disorder [8]. To verify this point, the energetic disorder in three OSCs is quantized by a parameter of Urbach energy. As shown in Fig. S11, the PBDB-TCl : AITC : BTP-eC9 film shows lower energetic disorder with an *E*_U_ of 22.04 meV than that of PBDB-TCl : AITC (23.74 meV) and PBDB-TCl : BTP-eC9 (22.67 meV). The variation of *E*_U_ is consistent with that of Δ*V*_2_, which confirms that the lower energetic disorder (Fig. 3f) reduces Δ*V*_2_ in ternary OSCs.

To gain further insight into the composition of *V*_loss_, EQE_EL_ measurements of three devices are performed. As shown in Fig. 3g, the EQE_EL_ values are 1.01 × 10^−5^ and 3.25 × 10^−4^ for the PBDB-TCl : AITC and PBDB-TCl : BTP-eC9 binary OSCs, corresponding to Δ*V*_3_ of 0.34 and 0.21 V, respectively. For the ternary device, the EQE_EL_ is measured as 7.68 × 10^−4^, corresponding to Δ*V*_3_ of 0.19 V. Therefore, compared with the PBDB-TCl : BTP-eC9 OSCs, the higher *V*_OC_ of ternary OSC with similar *E*_g_ (1.38 eV) can be attributed to both mitigations in Δ*V*_3_ (from 0.21 to 0.19 V) and Δ*V*_2_ (from 0.06 to 0.05 V). The details of representative and efficient OSCs are summarized in Fig. 3i. In this work, the photovoltaic parameters of ternary OSCs are located in the overlap region with simultaneously high PCE and low Δ*V*_loss_. The above results indicate that pursuing high *V*_OC_ while maintaining the *J*_SC_ and FF values is still one of the most effective ways towards achieving high-performance OSCs.

### Analysis of film morphology

**Figure 4.**
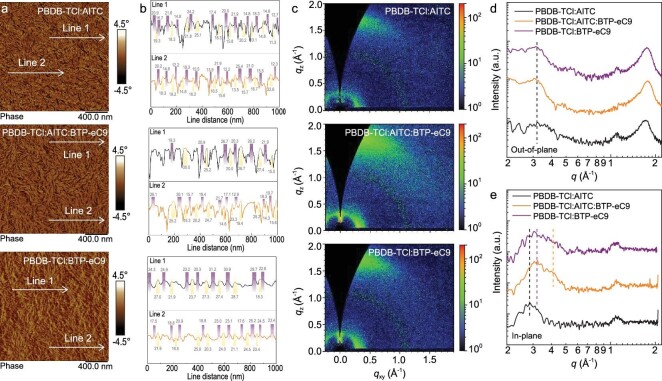
Morphology of thin films. (a) The atomic force microscopy (AFM) phase images of binary and ternary blended films. (b) Line profiles to obtain the full-width at half-maximum (FWHM) of cross-section of the fibral through AFM phase images. (c) 2D grazing incidence wide angle X-ray scattering (GIWAXS) patterns of the binary and ternary films. The *q*_xy_ and *q*_z_ corresponding scattering vector in the in-plane (IP) and out-of-plane (OOP) directions. (d and e) OOP and IP line-cut profiles of the 2D GIWAXS patterns based on binary and ternary blended films.

To obtain more insights into the variation of photovoltaic performances in the ternary OSCs, the surface textures and aggregation of films are investigated by AFM. As shown in Fig. 4a and Fig. S12, PBDB-TCl : AITC, PBDB-TCl : AITC : BTP-eC9 and PBDB-TCl : BTP-eC9 films exhibit surface roughness (*R*_q_) of 1.84, 1.95 and 2.76 nm, respectively. The detailed phase separation of the films was then analyzed through the AFM phase images. Figure 4b shows the line profile FWHM of the peaks in the corresponding AFM phase images (Figure 4a). The derived average values of inter-fibril distance are 17.5, 20.1 and 23.4 nm for the PBDB-TCl : AITC, PBDB-TCl : AITC : BTP-eC9 and PBDB-TCl : BTP-eC9 films, respectively. In addition, the power spectral density (PSD) analysis is conducted on the blend films to investigate quantitative information regarding domain properties [42,43]. The 1D PSD × *q*^2^ profiles as a function of spatial frequency (*q*) from the AFM phase images of the blends are provided in Fig. S13. It can be seen that the addition of AITC reduces the peak location of 1D-PSD(*q*), which means a decreased domain size. These results reveal that the introduction of AITC influences the molecular aggregation and phase separation of the ternary active layer.

GIWAXS is utilized to investigate the molecular orientation and crystallinity in the active layer thin films. First, the 2D GIWAXS patterns and corresponding 1D line cut profiles of neat films are shown in Figs. S14 and 15 and Table S7 in SI. All the films show face-on molecular orientations that are evidenced by the strong reflection of π–π stacking in the out-of-plane (OOP) directions. We further use Gaussian functions to differentiate and analyze the π–π stacking peaks, in order to obtain the information for peak area, FWHM and derived crystal coherence length (CCL) associated with the π–π stacking [44]. The face-on orientations with π–π stacking peaks at 1.70 Å^−1^ (*d*-spacing : 3.70 Å), 1.79 Å^−1^ (*d*-spacing : 3.50 Å) and 1.85 Å^−1^ (*d*-spacing : 3.40 Å) are observed for the PBDB-TCl, BTP-eC9 and AITC films, respectively. By fitting the π–π stacking peaks of all the neat films, the AITC film shows a CCL of 2.21 nm as opposed to that of PBDB-TCl (1.68 nm) and BTP-eC9 (1.96 nm), which is beneficial to the increased CCL in PBDB-TCl : AITC and PBDB-TCl : AITC : BTP-eC9 blends.

As shown in Fig. 4c–e, 2D GIWAXS and corresponding line-cut profiles of the blended films show the peaks near 0.30 Å^−1^ in the OOP directions, which are assigned to the diffraction of PBDB-TCl lamellar. In the IP directions, the peak near 0.30 Å^−1^, 0.33 Å^−1^ and 0.40 Å^−1^ are assigned to the diffraction of PBDB-TCl, AITC and BTP-eC9, respectively (Fig. S16 and Table S8). Moreover, PBDB-TCl : AITC, PBDB-TCl : AITC : BTP-eC9 and PBDB-TCl : BTP-eC9 films show the π–π stacking located at 1.72 Å^−1^,1.77 Å^−1^ and 1.75 Å^−1^, respectively, corresponding to *d*-spacings of 3.65 Å, 3.54 Å and 3.59 Å (Fig. S17 and Table S9). The larger film thickness normalized-peak area (1.58) and CCLs (2.28 nm) than that of PBDB-TCl : BTP-eC9 film (1.23 and 2.22 nm) indicates that the introduction of AITC can reinforce molecular packing along with increased crystal size in PBDB-TCl : AITC : BTP-eC9 films. In addition, we analyze the relative population of crystallites of face-on and edge-on orientations relative to the substrate. The areas integrated with polar angle (*θ*) ranges of 0–45° (A_1_) and 45–90° (A_2_) are defined as the portion of edge-on and face-on crystallites, respectively, and the ratio of A_2_/A_1_ is regarded as the face-on to edge-on ratio [45]. As shown in Fig. S18, the A_2_/A_1_ of PBDB-TCl : AITC, PBDB-TCl : AITC : BTP-eC9 and PBDB-TCl : BTP-eC9 are 3.52, 5.16 and 4.65, respectively, which indicates that the introduction of AITC can increase the population of face-on crystallites of ternary film. These results account for improved and balanced carrier transport, suppressed charge recombination, decreased Δ*V*_loss_ and improved device photovoltaic performance.

### Performance of tandem OSCs

**Figure 5.**
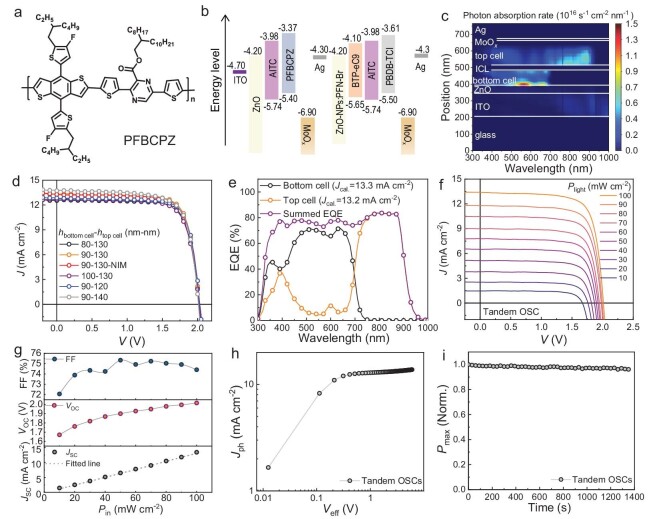
Photovoltaic performance of tandem OSCs. (a) Chemical structure of wide-bandgap polymer PFBCPZ. (b) Energy level diagram of the components used to fabricate tandem OSCs. (c) Distributions of photon absorptions of the tandem OSCs with glass/ITO structure (150 nm)/ZnO (30 nm)/PFBCPZ : AITC (90 nm)/MoO_3_ (7 nm)/Ag (0.5 nm)/ZnO-NPs : PFN-Br (20 nm)/PBDB-TCl : AITC : BTP-eC9 (130 nm)/MoO_3_(7 nm)/Ag (120 nm). (d) *J-V* curves of tandem OSCs with varying thickness of the active layer. (e) EQE spectra of optimal tandem OSC. (f and g) Light-intensity dependent *J*_SC_ and *V*_OC_ characteristics of the optimal tandem OSCs. (h) Photocurrent density (*J*_ph_) versus effective bias (*V*_eff_) for the tandem OSCs. (i) MPP tracking profile of the tandem OSCs.

To explore the application potential of ternary active layers, the tandem devices are further fabricated. First, the wide bandgap polymer PFBCPZ is selected to blend with AITC as a bottom sub-cell active layer material to ensure the ideal match of current with top sub-cell [24,46]. The molecular structure, energy level and film absorbance spectra of PFBCPZ are shown in Fig. 5a and b and Fig. S19. The detailed photovoltaic parameters of the PFBCPZ : AITC-based single-junction OSCs are provided in Fig. S19 and Table S10. PFBCPZ : AITC OSCs exhibit a high *V*_OC_ of 1.17 V, which is beneficial to achieving high photovoltaic parameters in tandem OSCs. The tandem OSCs are constructed by stacking inverted sub-cells (Fig. 5c). Subsequently, the optical field distribution within tandem devices is investigated by using the transfer-matrix modeling method [47]. As shown in Fig. 5c, the photon absorption rate distribution in the bottom sub-cell is primarily located in the wavelength range of 300–700 nm. The top cell absorbs radiation primarily located in the infrared region, at wavelengths of 700–1000 nm. Moreover, based on the optical distribution simulation and its integration over wavelength (Fig. S21a), a balanced and maximal theoretical value of over 18.9 mA cm^−2^ is obtained when the optimal thicknesses of the active layers in the bottom and top sub-cells are 90 and 150 nm (Fig. S21b), respectively.

Next, tandem OSCs were fabricated, and their photovoltaic performances are determined by measuring *J−V* curves under AM1.5 G, 100 mW cm^−2^ illumination. As shown in Fig. 5d and Table 1, the tandem OSCs produce a maximal current density of 13.7 mA cm^−2^ when the active layer thicknesses of the bottom and top sub-cells are controlled to 90 and 140 nm, which is almost identical with above simulations. By optimizing the thickness of the active layer in detail, the tandem OSCs containing active layer thicknesses of 90 and 130 nm in the bottom and top sub-cells, respectively, exhibit a *V*_OC_ of 2.02 V, *J*_SC_ of 13.3 mA cm^−2^, FF of 76.6% and a PCE of 20.6% (verified as 20.3% by NIM, China, as shown in Fig. S22 and Table 1), which is the highest value for OSCs. The top sub-cell containing the ternary active layer shows a broad EQE spectrum with a high response <80% in the range of 750–900 nm. The *J*_SC_ values of tandem OSCs are verified by the calculated current density (*J*_cal._) from the EQE spectra in Fig. 5e. The *J*_cal._ values of bottom and top sub-cells are 13.3 and 13.2 mA cm^−2^, respectively, indicating the highly balanced current generation in each sub-cell. Besides, the *J*_cal_’s obtained by EQE spectra are similar to the *J*_SC_ derived from *J−V* curves (deviations lower than 1%), which further confirm that the measurements are accurate.

To further investigate the charge recombination and photoelectrical properties of the tandem OSCs, the *J−V* curves of the device are measured under various light intensities (*P*_light_), and the results are shown in Fig. 5f. As shown in Table S12, the PCEs of the tandem OSCs remain at 17.8% when the *P*_light_ varies from 100 to 10 mW cm^−2^. Moreover, the *J*_SC_ of the tandem OSCs follows a power-law dependence with respect to *P*_light_ (Fig. 5g), and the slope value is 0.97, which suggests no substantial space charge build-up in both the two sub-cells and the interconnecting layer of the tandem OSCs [48]. The dependence of *V*_OC_ and FF on *P*_light_ are shown in Fig. 5g, respectively. The FF of the tandem devices increases under low light intensity (50 mW cm^−2^), which is due to weak charge recombination in the active layer. The *V*_OC_ increases with the light intensity, which is similar to that in other reported single-junction OSCs. Clearly, these results indicate that the superior tandem OSCs are constructed by using two well performing sub-cells and an effective interconnecting layer. The charge dissociation property of tandem OSCs is studied by measuring the relationship between the photocurrent density and effective bias. As shown in Fig. 5h, the photocurrents are saturated at large reverse biases, which indicates that all the charge carriers are dissociated and collected at the electrodes. Consequently, the *P*_diss_ of tandem OSCs is determined to be 95.3%. As the overall *P*_diss_ of the tandem cell is much larger than the reference organic solar cell, it suggests that the intrinsic *P*_diss_ of the organic sub-cell is improved. Then, the operational stability of the tandem OSCs is also investigated under continuous light illumination by using maximum power point (MPP) tracking measurements. Figure 5i shows the time dependence of the normalized PCE of the devices. The best tandem OSC shows 4% degradation of initial PCE within 1380 s exposure. These results illustrate that PBDB-TCl : AITC : BTP-eC9–based tandem OSCs exhibit outstanding operational stability.

## CONCLUSIONS

Here, a tandem OSC with 20.6% PCE is achieved, which is featured by a Δ*V_loss_-*restraining multicomponent active layer containing polymer PBDB-TCl and two small molecule acceptors AITC and BTP-eC9. Benefiting from the unique morphology of the ternary active layer, efficient charge generation, improved carrier migration and reduced voltage loss (0.50 V) are realized. Hence the PBDB-TCl : AITC : BTP-eC9 ternary OSCs achieved a PCE of 19.1% (certified as 18.9%) by simultaneously increasing the open-circuit voltage, short-circuit current and fill factor. In addition, the tandem OSCs based on the PFBCPZ : AITC bottom active layer and the PM6 : AITC : BTP-eC9 top active layer yielded a record PCE of 20.6% (certified as 20.3%), which is the highest PCE in OSCs field. Overall, this work not only reports an outstanding PCE but also demonstrates that decreasing the voltage losses, and adopting ternary and tandem device structures are effective strategies for further improving the efficiency of the OSCs.

## MATERIALS AND METHODS

### Materials

The synthesis and purification of PFBCPZ and AITC were reported in our previous report [24,46]. PBDB-TCl, BTP-eC9, PNDIT-F3N-Br were purchased from Solarmer Materials Inc. and used as received. PEDOT : PSS (clevios P VP Al 4083) was purchased from H.C. Starck Co. Ltd. The glass/ITO substrates were purchased from Huananxiangcheng Inc.

### Fabrication of single-junction OSCs

Glass/indium-tin-oxide (ITO) substrates were sequentially cleaned in detergent, deionized water, acetone and isopropanol, respectively. ITO substrates were treated with ultraviolet-ozone (UVO) for 15 min, and then PEDOT : PSS solution was spin-coated on the ITO substrates. The PEDOT : PSS-coated ITO substrates were annealed at 160°C for 15 min under air atmosphere. Active layers were obtained by spin-coating the blend solutions on the PEDOT : PSS layers with a thickness of ∼100 nm. Active layer materials were dissolved in chloroform at a polymer concentration of 7.5 mg mL^−1^. The weight ratio of donor to acceptor was fixed at 1 : 1.2 for PBDB-TCl : AITC, PBDB-TCl : BTP-eC9, and PFBCPZ : AITC and at 1 : 0.2 : 1.2 for PBDB-TCl : AITC : BTP-eC9. All the solutions were stirred at 40°C for 4 h. Before spin-coating the active layer, 0.5% 1,8-diiodooctane (v/v) was added to the solutions. The active layers were annealed at 100°C for 10 min. Then, a 5 nm-thick layer of PNDIT-F3N-Br was spin-coated on the top of the active layers. Finally, a 150 nm layer of Ag was deposited by thermal evaporation through shadow masks, leading to a defined active area of 0.09 cm^2^. The area of the aperture was 0.0625 cm^−2^.

### Fabrication of tandem OSCs

Glass/ITO substrates were cleaned and treated with UVO according to the abovementioned methods. PEDOT : PSS was spin-coated on top of ITO substrates, followed by 15 min annealing at 160°C. After annealing, the PEDOT : PSS coated ITO substrates were transferred into an N_2_ glove box. Then, the PFBCPZ : AITC solution was spin-coated, followed by annealing at 110°C for 10 min. Then, 7 nm MoO_x_ layer and 0.5 nm ultra-thin Ag layer were evaporated under high vacuum. Subsequently, ZnO : PFN-Br solution was cast on the bottom cells and annealed at 110°C for 30 min. After that, the solution of PBDB-TCl : AITC : BTP-eC9 was spin-coated to obtain films with different thicknesses, and then the active layers were thermally annealed at 110°C for 10 min. Finally, 7 nm MoO_x_ and 120 nm Ag layers were thermally evaporated under high vacuum (3 × 10^−4^ Pa). The area of cells, defined by the overlap between the ITO and Ag electrodes, was 0.037 mm^2^. The area of the aperture was 0.0225 cm^2^.

## Supplementary Material

nwad085_Supplemental_File
