# Supplementary material for: Tandem organic solar cells with 20.6% efficiency enabled by reduced voltage losses
Source: Natl Sci Rev. 2023 Mar 30;10(6):nwad085. doi: 10.1093/nsr/nwad085 (PMC10337743; doi:10.1093/nsr/nwad085)
Supplement: nwad085_Supplemental_File [file nwad085_supplemental_file.docx]

SUPPLEMENTARY INFORMATION

**Tandem organic solar cells with 20.6% efficiency enabled by reduced voltage losses**

Jianqiu Wang,^1,2^ Zhong Zheng,^1,3^* Pengqing Bi,^1^ Zhihao Chen,^1,4^ Yafei Wang,^1,6^ Xiaoyu Liu,^1,3^ Shaoqing Zhang,^1,3^ Xiaotao Hao,^4^ Maojie Zhang,^2^* Yongfang Li,^2,5,6^ and Jianhui Hou^1,3,6^*

^1^State Key Laboratory of Polymer Physics and Chemistry, Beijing National Laboratory for Molecular, Institute of Chemistry Chinese Academy of Sciences, Beijing 100190, China.

^2^Laboratory of Advanced Optoelectronic Materials, Suzhou Key Laboratory of Novel Semiconductor-optoelectronics Materials and Devices, College of Chemistry, Chemical Engineering and Materials Science, Soochow University, Suzhou 215123, China.

^3^School of Chemistry and Biology Engineering University of Science and Technology Beijing, Beijing 100083, China.

^4^School of Physics, State Key Laboratory of Crystal Materials, Shandong University, Jinan, 250100, China

^5^CAS Key Laboratory of Organic Solids, Beijing National Laboratory for Molecular Sciences, Institute of Chemistry, Chinese Academy of Sciences, Beijing 100190, China.

^6^University of Chinese Academy of Sciences, Beijing 100049, China.

*Corresponding authors: zhongzheng@ustb.edu.cn; [mjzhang@suda.edu.cn](mailto:mjzhang@suda.edu.cn); [hjhzlz@iccas.ac.cn](mailto:hjhzlz@iccas.ac.cn)

**Sample characterizations**

Ultraviolet photoelectron spectroscopy (UPS) was performed on Thermo Scientific ESCALab 250Xi. The gas discharge lamp was used for UPS, with Helium gas admitted and the He I emission line at 21.22eV employed. The Helium pressure in the analysis chamber during analysis is about 3×10^-8^ mbar. Hitachi UH5300 spectrophotometer was used to measure the absorption spectra. The *J-V* characteristics of the devices were measured under AM1.5G illumination, 100 mW cm^-2^. The light intensity was calibrated by the standard silicon solar cell. EQE spectra were measured by a system of QE-R3011 (Enlitech Technology Co., Ltd). To measure the EQE of bottom and top cell, light bias obtained by 500 nm short wave pass filters and 850 nm long wave pass filters are selected to excite the bottom and top cells, respectively. FTPS-EQE was measured using an integrated system (PECT-600, Enlitech) EQE_EL_ and EL spectra were collected through the devices (ELCT-3010, Enlitech). TA measurements were performed on an Ultrafast Helios pump-probe system in collaboration with a regenerative amplified laser system from Coherent. An 800 nm pulse with a repetition rate of 1k Hz, a length of 100 fs, and an energy of 7 mJ pluse^-1^, was generated by an Ti:sapphire amplifier (Astrella, Coherent). Then the 800 nm pulse was separated into two parts by a beam splitter. One part was coupled into an optical parametric amplifier (TOPAS, Coherent) to generate the pump pulses at various wavelengths. The other part was focused onto a sapphire plate and a YAG plate to generate white light supercontinuum as the probe beams with spectra covering 420-800 nm and 750-1300 nm, respectively. The time delay between pump and probe was controlled by a motorized optical delay line with a maximum delay time of 8 ns. The pump pulse is chopped by a mechanical chopper with 500 Hz and then focused onto the mounted sample with probe beams. The probe beam was collimated and focused into a fiber-coupled multichannel spectrometer with CCD sensor. The energy of pump pulse was measured and calibrated by a power meter (PM400, Thorlabs). Photo-CELIV mobilities, TPV data, Charge extraction data, Mott-Schottky data and *C-V* data were obtained by the all-in-one characterization platform, Paios (Fluxim AG, Switzerland). Atomic force microscopy (AFM) height and phased images were measured by a Bruker Nanoscope V AFM microscope. GIWAXS were measured on a Xeuss 2.0 SAXS/WAXS system (Xenocs SA, France). Cu Kα X-ray source (GeniX3D Cu ULD), generated at 50 kV and 0.6 mA, was utilized to produce X-ray radiation with a wavelength of 1.5418 Å. A semiconductor detector (Pilatus 300 K, DECTRIS, Swiss) with a resolution of 487*619 pixels was used to collect the scattering signals. The incident angle is 0.2°. Spectra ellipsometer (SENTECH-850) was used to collect the optical parameters of the films. The spectral range is from 300 nm to 1050 nm. The incidence angle range is from 0 to 90°. The optical constant of the materials can be obtained from the ellipsometry data, by performing a weighted least-squares regression analysis method (Levenberg-Marquardt algorithm).

**Calculation methods**

**Determinations of energy level and work function**

The highest occupied molecular orbitals (HOMO) and work function are calculated from the spectrum obtained by UPS. The secondary electron onset value (*E*_cutoff_), the valence band maximum value (*E*_VBM_), and the energy of He I (*h*ν) was used to calculate the ionization potential (IP, IP value is the inverse of HOMO value) by applying the Equation S1.

*E*_HOMO_ = -IP = -(*Φ* + *E*_VBF_) = -(*hv* - *E*_cutoff_ +*E*_VBM_) Equation S1

*E*_cutoff_ is obtained by onset point from the cross point between two trend lines (one is placed on the baseline and the other on the slope of the peak). The Fermi energy level (*E*_F_) of neat films was calculated by follow equation,

*E*_F_ = *hν* − (*Ε*_F__,Au_ − *Ε*_cutoff_) Equation S2

where *Ε*_F,Au_ is the Fermi energy level (*E*_F_) of gold. *Ε*_F,Au_ is 20.97 eV in this work. Optical gaps (*E*_g,opt_) were determined from the onset of UV-visible absorption spectra using a procedure similar to the determinations of UPS onsets. The *E*_g,opt_ corresponds to the lowest gap between the lowest occupied molecular orbital band (LUMO) and HOMO.

**Determinations of voltage losses**

The voltage losses in photovoltage cell can be categorized into three contributions:

$\text{∆V=}\frac{\text{E}_{\text{g}}}{\text{q}}\text{-}\text{V}_{\text{oc}}$= ($\frac{\text{E}_{\text{g}}}{\text{q}}\text{-}\text{V}_{\text{OC}}^{\text{SQ}}$)+($\text{ }\text{V}_{\text{OC}}^{\text{SQ}}\text{-}\text{V}_{\text{OC}}^{\text{rad}}$)+($\text{ }\text{V}_{\text{OC}}^{\text{rad}}\text{-}\text{V}_{\text{OC}}$) = Δ*V*_1_+Δ*V*_2_+Δ*V*_3_ Equation S3

The E_g_ value is determined by the derivatives of the Fourier transform photocurrent spectroscopy (EQE_FTPS_) curve and then calculated with the following equation:

$\text{E}_{\text{g}}\text{=}\frac{\int_{\text{a}}^{\text{b}} \text{E}_{\text{g}}\text{P(}\text{E}_{\text{g}}\text{)d}\text{E}_{\text{g}}}{\int_{\text{a}}^{\text{b}} \text{P(}\text{E}_{\text{g}}\text{)d}\text{E}_{\text{g}}}$ Equation S4

$\text{V}_{\text{OC}}^{\text{SQ}}$ is the maximum voltage based in the Shockley-Queisser (SQ) limit, where the EQE_PV_ is assumed to be a step-function, i.e., 1 above the gap and 0 below the gap. In SQ limit, $\text{V}_{\text{OC}}^{\text{SQ}}$ follows

$\text{V}_{\text{OC}}^{\text{SQ}}\text{=}\frac{\text{KT}}{\text{q}}\ln\left( \frac{\text{J}_{\text{SC,SQ}}}{\text{J}_{\text{0,SQ}}}\text{+1} \right)\text{=}\frac{\text{KT}}{\text{q}}\ln\left( \frac{\text{q}\int_{\text{Eg}}^{\text{∞}} \text{∅}_{\text{AM1.5}}\text{(E)dE}}{\text{q}\int_{\text{Eg}}^{\text{∞}} \text{∅}_{\text{bb}}\text{(E)dE}}\text{+1} \right)$ Equation S5

where $\text{∅}_{\text{AM1.5}}$ is the solar radiation photo flux, $\text{∅}_{\text{bb}}$ is a black body radiation at 300K. $\text{V}_{\text{OC}}^{\text{rad}}$ is the voltage where the all recombination is radiative (i.e., EQE_EL_=1), and follows from

$\text{V}_{\text{OC}}^{\text{rad}}\text{=}\frac{\text{KT}}{\text{q}}\ln\left( \frac{\text{J}_{\text{SC,rad}}}{\text{J}_{\text{0,rad}}}\text{+1} \right)\text{=}\frac{\text{KT}}{\text{q}}\ln\left( \frac{\text{q}\int_{\text{Eg}}^{\text{∞}} {\text{EQE}_{\text{PV}}\text{∅}}_{\text{AM1.5}}\text{(E)dE}}{\text{q}\int_{\text{Eg}}^{\text{∞}} {\text{EQE}_{\text{PV}}\text{∅}}_{\text{bb}}\text{(E)dE}}\text{+1} \right)$ Equation S6

The voltage loss due to non-radiative recombination can be obtained by:

$\text{∆V}_{\text{OC}}^{\text{non-rad}}\text{=}\text{V}_{\text{OC}}^{\text{rad}}\text{-}\text{V}_{\text{OC}}$ Equation S7

**Determinations of trap DOS**

The defects density can be calculated form capacitance-frequency spectroscopy measurement in dark environment. The frequency axis can be scaled to energy axis through the follows

$\text{E}_{\text{ω}}\text{ =}\text{kT}\ln\left( \frac{\text{2}\text{v}_{\text{0}}}{\text{ω}} \right)$ Equation S8

where 𝜔 is the angular frequency calculated by 𝜔 = 2𝜋𝑓, 𝜈_0_ is the attempt-to-escape frequency of 10^9^ Hz [1]. The trap density at energy 𝐸_t_(E𝜔) can be acquired as

$\text{E}_{\text{t}}\left( \text{E}_{\text{ω}} \right)\text{=-}\frac{\text{V}_{\text{bi}}}{\text{qd}}\frac{\text{d}\text{C}}{\text{d}\text{ω}}\frac{\text{ω}}{\text{kT}}$ Equation S9

*d* is the thickness of the active layer and *V*_bi_ is the built-in voltage measured through Mott–Schottky characterization (shown in Fig. S8). Then the energy distribution can be described with Gaussian shape distribution

$\text{N}_{\text{t}}\text{(}\text{E}\text{)=}\frac{\text{N}_{\text{t}}}{\sqrt{\text{2π}}\text{σ}}\left[ \text{-}\frac{{\text{(}\text{E}_{\text{t}}\text{-}\text{E}\text{)}}^{\text{2}}}{\text{2}\text{σ}^{\text{2}}} \right]$ Equation S10

where *N*_t_ is the total density, *E*_t_ is the center of the DoS, and *σ* is the disorder parameter.


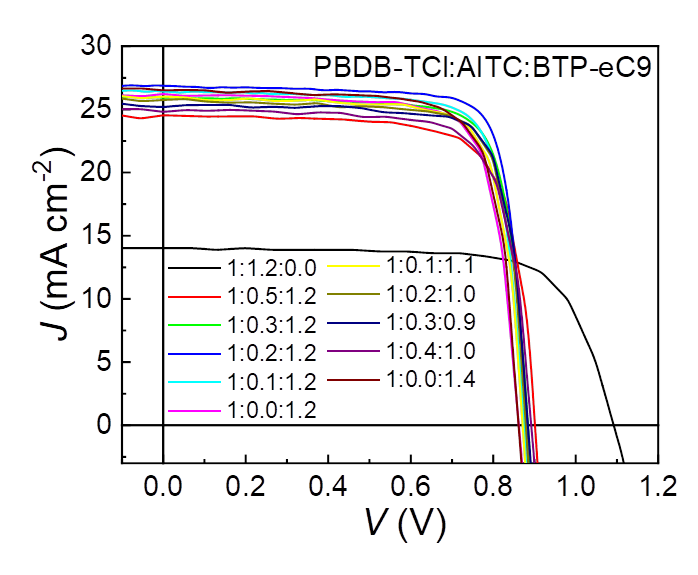


**Fig. S1**. *J*-*V* curves of OSCs based on PBDB-TCl:AITC:BTP-eC9 active layer with various ratio of donor and acceptor.

**Table S 1.** Photovoltaic parameters of PBDB-TCl:AITC:BTP-eC9 based OSCs with various ratio of donor and acceptor.

| PBDB-TCl:AITC:BTP-eC9 | *V*_OC_ (V) | *J*_SC_ (mA cm^-2^) | FF (%) | PCE (%) |
| --- | --- | --- | --- | --- |
| 1:1.2:0.0 | 1.09 | 14.0 | 72.6 | 11.1 (10.8±0.3) |
| 1:0.5:1.2 | 0.90 | 24.5 | 74.2 | 16.4 (15.9±0.5) |
| 1:0.3:1.2 | 0.88 | 25.9 | 78.3 | 18.6 (18.1±0.4) |
| 1:0.2:1.2 | 0.88 | 26.9 | 80.5 | 19.1 (18.7±0.2) |
| 1:0.1:1.2 | 0.87 | 26.4 | 78.4 | 18.0 (17.6±0.3) |
| 1:0.0:1.2 | 0.86 | 26.2 | 76.5 | 17.2 (16.8±0.3) |
| 1:0.1:1.1 | 0.87 | 26.0 | 77.3 | 17.5 (17.0±0.4) |
| 1:0.2:1.0 | 0.88 | 25.7 | 78.0 | 17.7 (17.2±0.3) |
| 1:0.3:0.9 | 0.88 | 25.2 | 79.3 | 17.7 (17.1±0.5) |
| 1:0.4:0.8 | 0.89 | 24.8 | 75.2 | 16.6 (16.2±0.3) |
| 1:0.0:1.4 | 0.86 | 26.5 | 76.0 | 17.3 (16.9±0.3) |


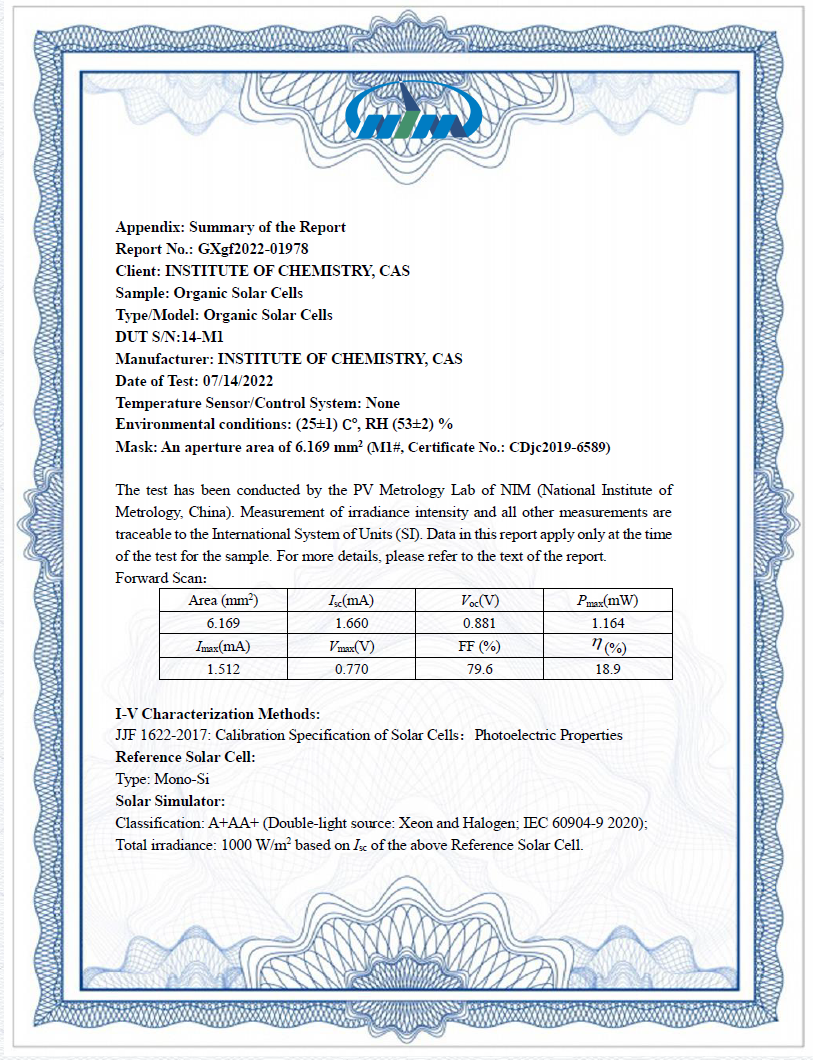


**Fig. S2.** The certification report of the best performance ternary cell based on PBDB-TCl:AITC:BTP-eC9 processed from NIM, China.


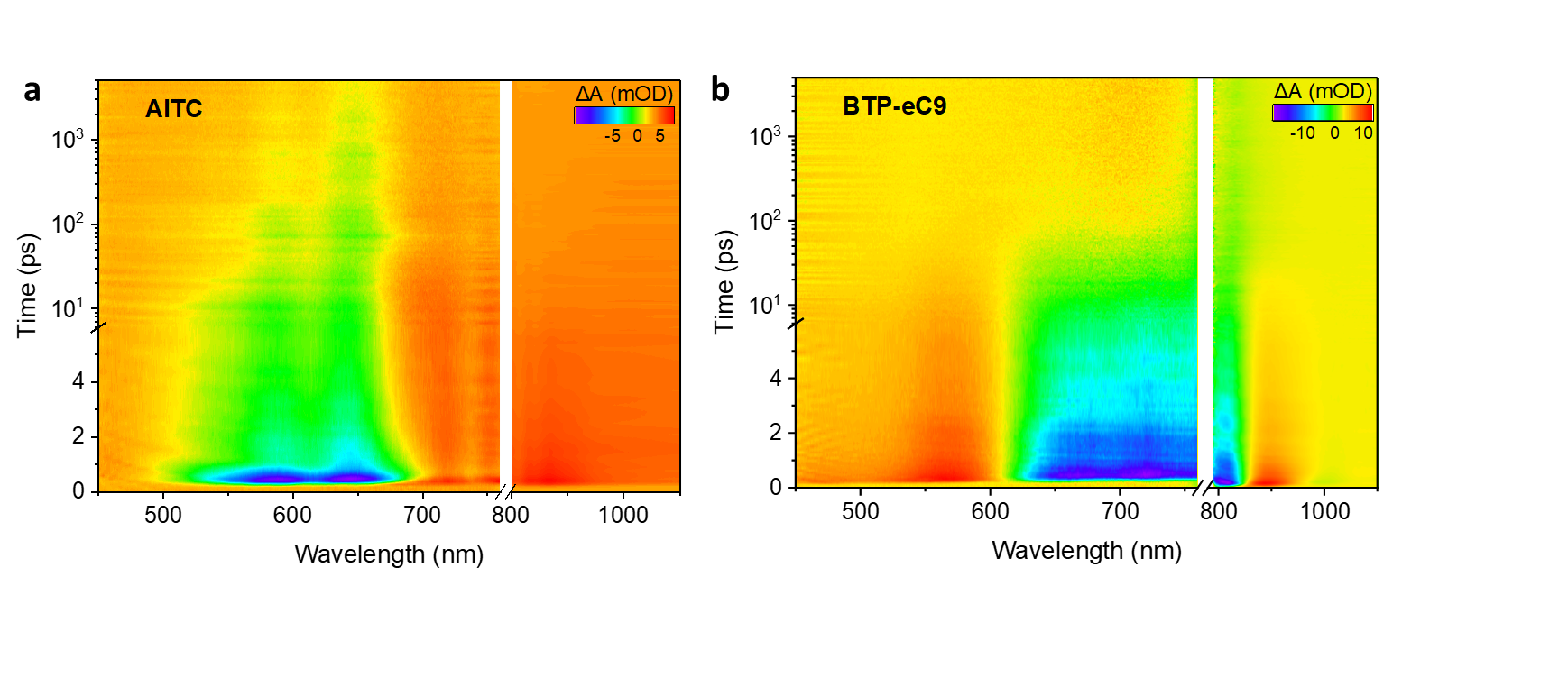


**Fig. S3.** 2DTA spectra of (a) PBDB-TCl:AITC:BTP-eC9 and (b) PBDB-TCl:BTP-eC9 under 660 nm and 800 nm excitation.


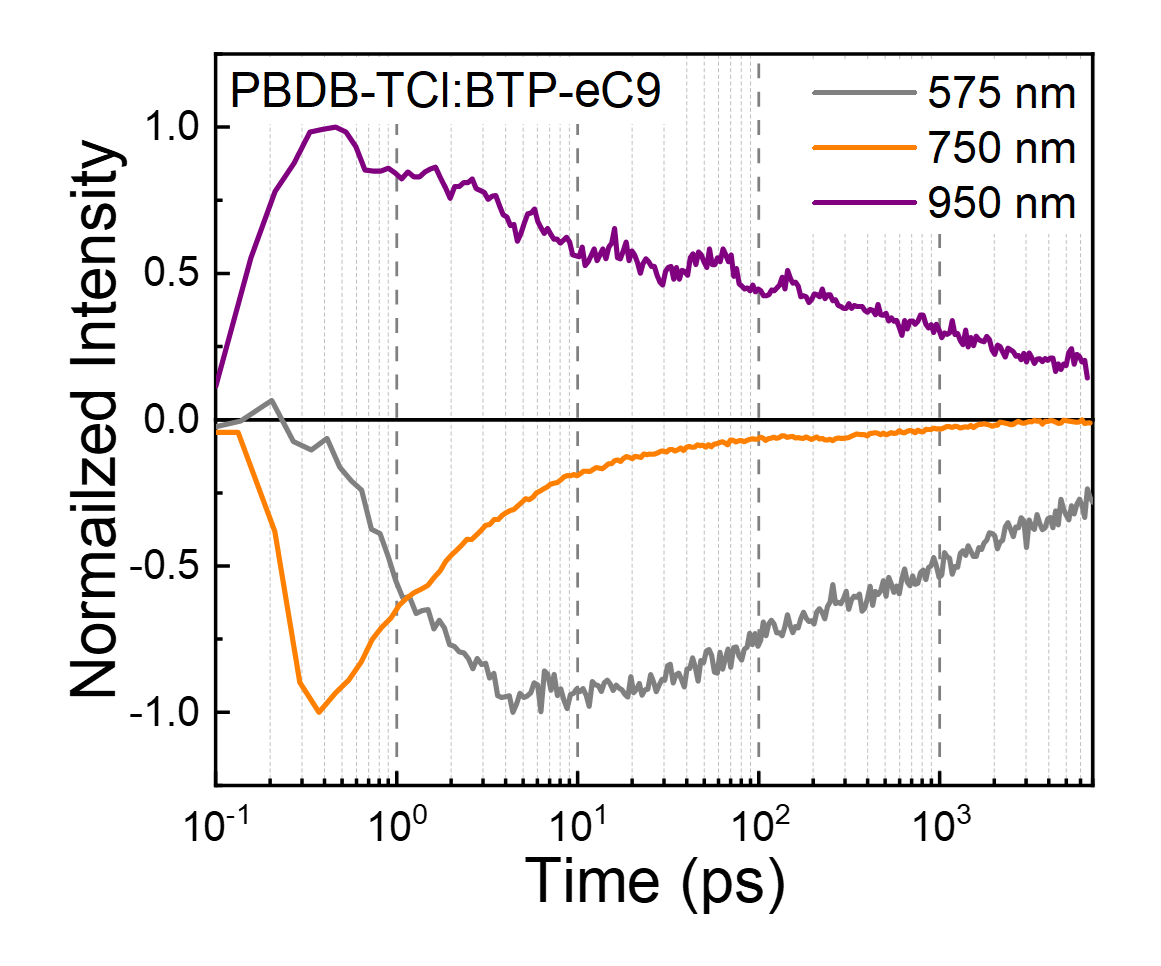


**Fig. S4.** Decay dynamics probed at 575, 750 and 950 nm in PBDB-TCl:BTP-eC9 systems under 800 nm excitation.

**Table S 2.** Time of the hole transfer process in blended films. The data was achieved through biexponential fitting.

| Active layer | *τ*_1_ (ps) | *τ*_2_ (ps) |
| --- | --- | --- |
| PBDB-TCl:AITC:BTP-eC9 | 0.05 | 0.74 |
| PBDB-TCl:BTP-eC9 | 0.62 | 1.04 |


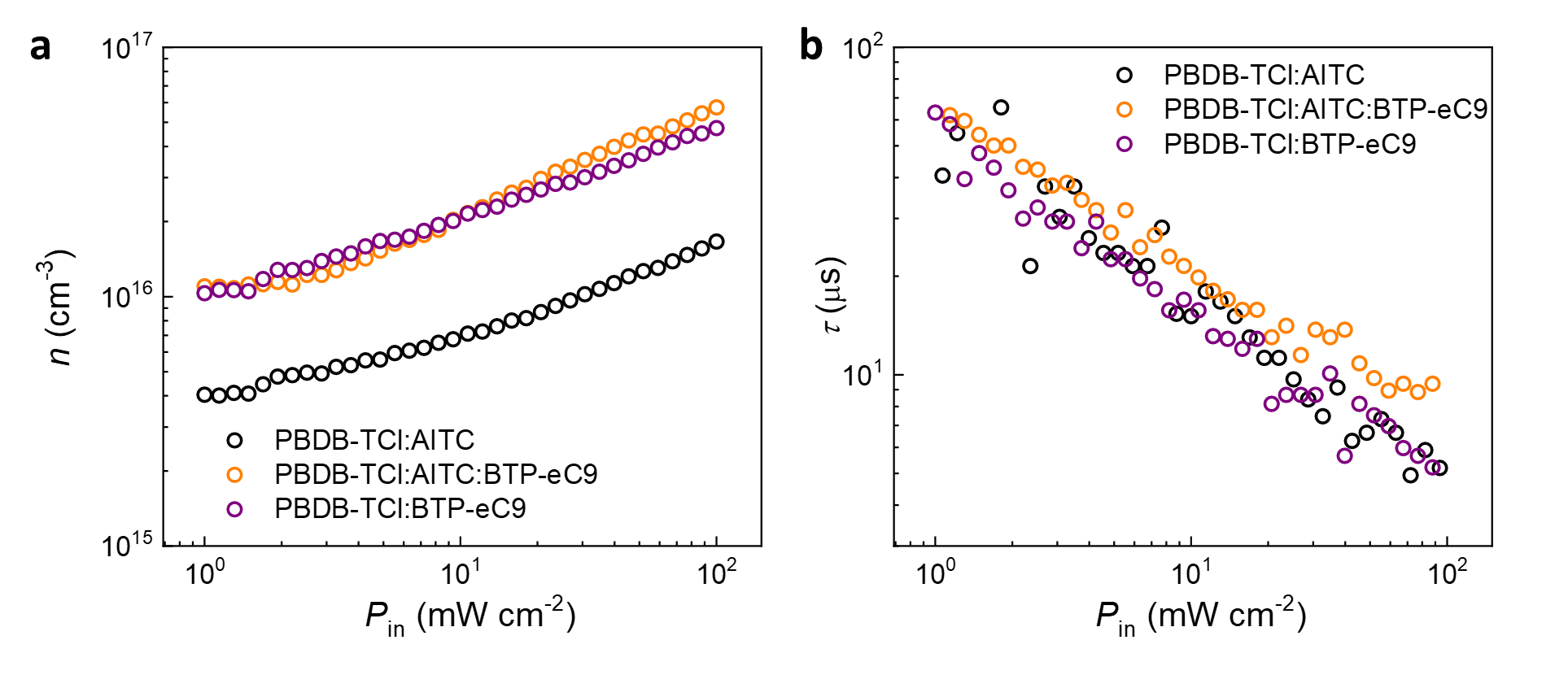


**Fig. S5.** (a) charge carrier density (*n*) and (b) charge carrier lifetime (*τ*) under various light intensity for three OSCs.


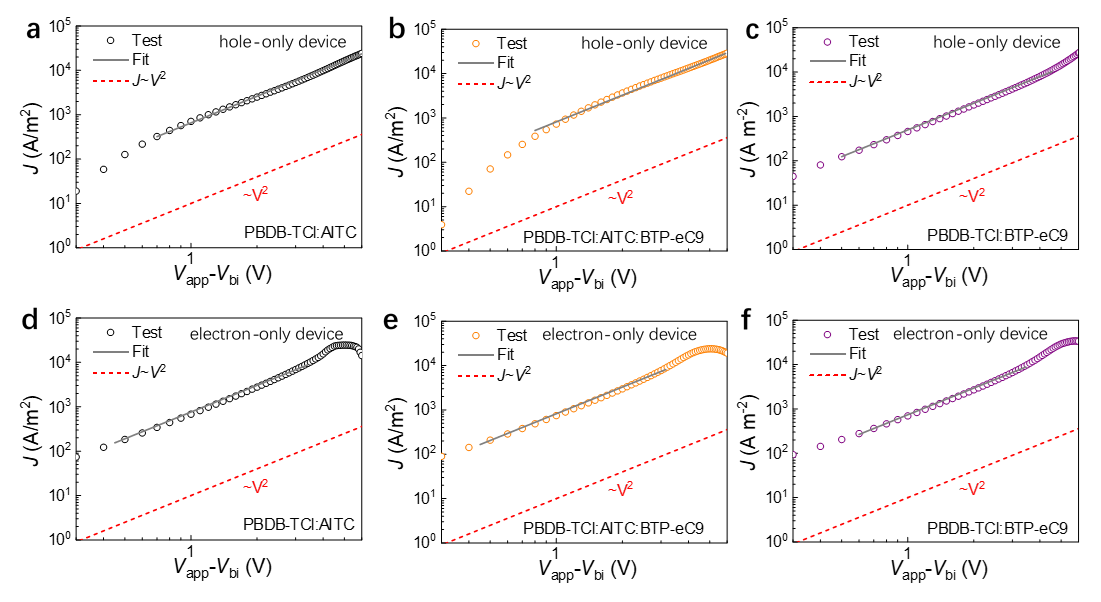


**Fig. S6.** Dark *J-V* curves of (a-c) hole-only and (d-f) electron-only devices containing different BHJ.

**
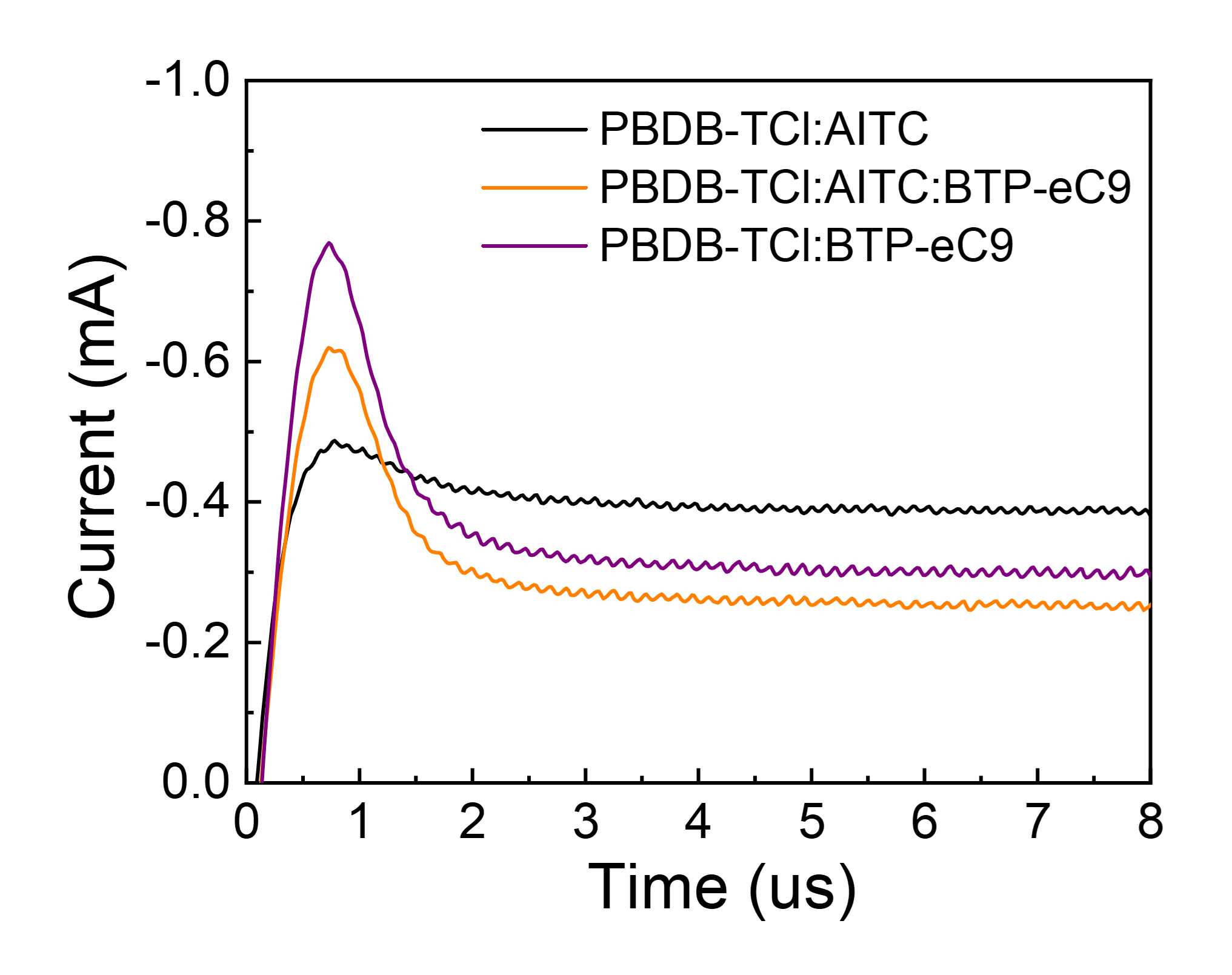
**

**Fig. S7.** Photo-CELIV curves of three OSCs. The ramp rate is 316 V/ms.

**Table S3.** The mobility of blend films.

| Active layer | *μ*_Hole_  (cm^2^ V^−1^ s^−1^) | *μ*_Electron_  (cm^2^ V^−1^ s^−1^) | *μ*_Bulk_  (cm^2^ V^−1^ s^−1^) | *μ*_Hole_/*μ*_Electron_ | Thickness (nm) |
| --- | --- | --- | --- | --- | --- |
| PBDB-TCl:AITC | 3.01×10^−4^ | 3.89×10^−4^ | 4.06×10^−4^ | 0.77 | 117 |
| PBDB-TCl:AITC:BTP-eC9 | 5.37×10^−4^ | 5.40×10^−4^ | 7.64×10^−4^ | 0.99 | 119 |
| PBDB-TCl:BTP-eC9 | 2.05×10^−4^ | 3.05×10^−4^ | 3.15×10^−4^ | 0.67 | 112 |

**
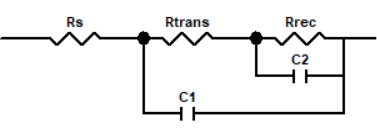
**

**Fig. S8.** Schottky equivalent circuit model used to fitting Nyquist plots.

**Table S4.** Transport and recombination resistance for three devices.

| OSCs | *R*_s_ (Ω) | *R*_trans_ (Ω) | *R*_rec_ (Ω) |
| --- | --- | --- | --- |
| PBDB-TCl:AITC | 31.7 | 261 | 1270 |
| PBDB-TCl:AITC:BTP-eC9 | 31.9 | 163 | 1872 |
| PBDB-TCl:BTP-eC9 | 39.0 | 281 | 1440 |


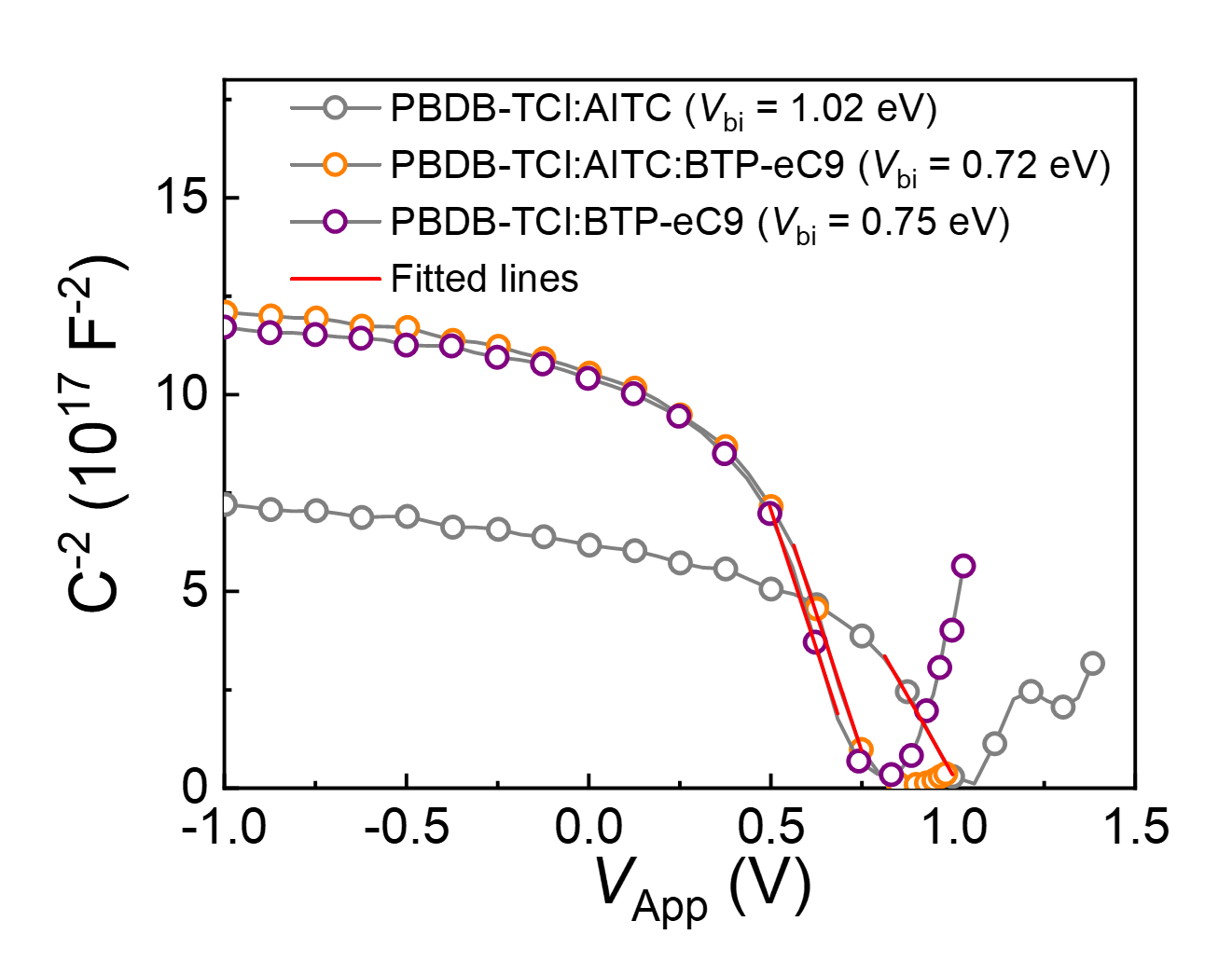


**Fig. S9.** Mott-Schottky characteristics of the OSCs measured at 10 kHz in dark to determine the built-in potential.

**Table S5.** Trap DOS fitting parameters for three OSCs.

| OSCs | *N*_t_ (10^18^ cm^-3^ eV^-1^) | *E*_t_ (eV) | *σ* (eV) |
| --- | --- | --- | --- |
| PBDB-TCl:AITC | 0.358 | 0.218 | 0.031 |
| PBDB-TCl:AITC:BTP-eC9 | 0.219 | 0.227 | 0.027 |
| PBDB-TCl:BTP-eC9 | 0.222 | 0.244 | 0.032 |


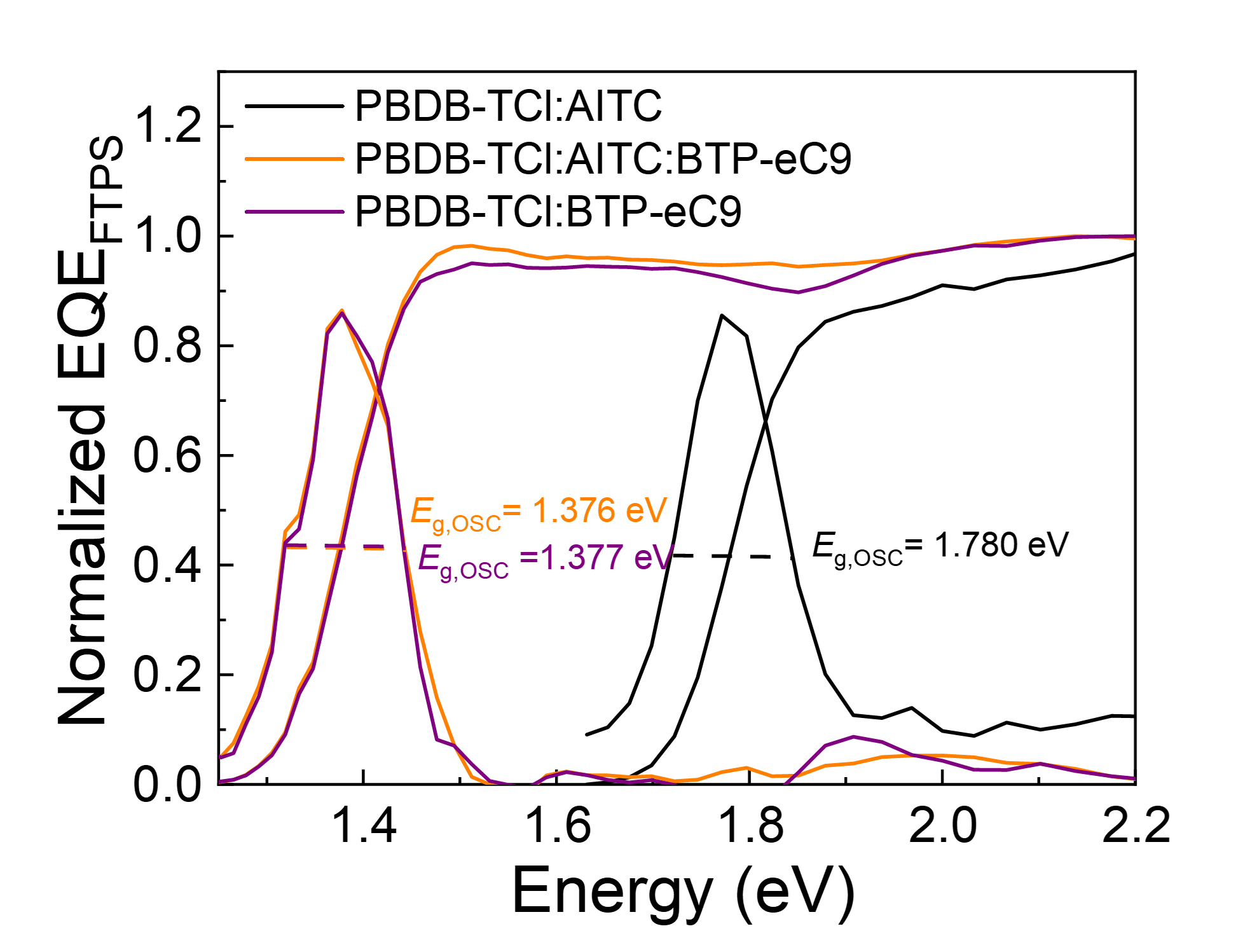


**Fig. S10.** Optical band gap (*E*_g,OSC_) distributions for three OSCs. *E*_g,OSC_ is the optical gap determined from the derivatives of the EQE_FTPS_ spectra.


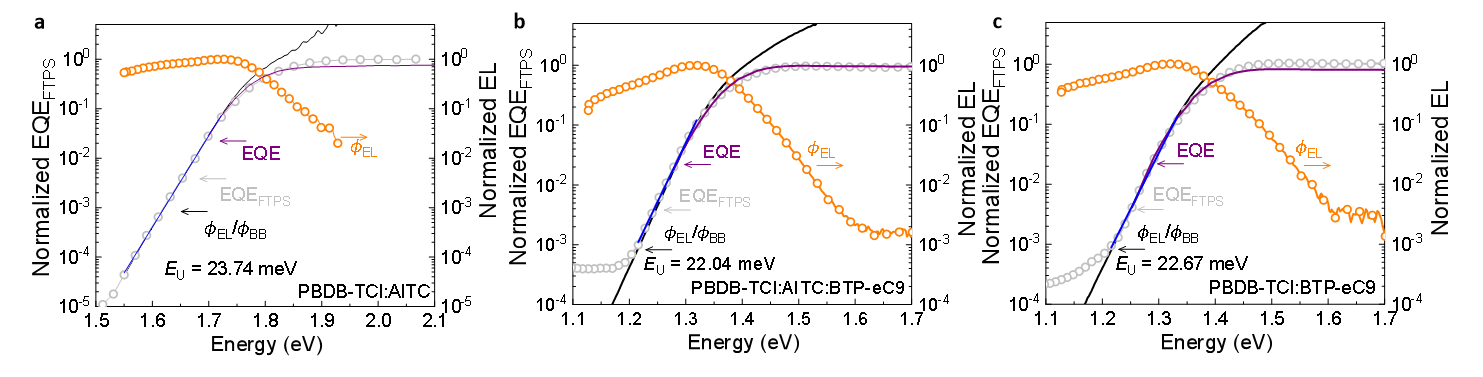


**Fig. S11** (a-c) The Fourier transform photocurrent spectroscopy (EQE_FTPS_) and electroluminescence (EL) spectra of binary and ternary OSCs. Urbach energy (*E*_U_) are calculated according to the equation $\text{α}\text{(}\text{E}\text{)=}\text{α}_{\text{0}}\text{e}^{\frac{\text{E}\text{-}\text{E}_{\text{0}}}{\text{E}_{\text{U}}}}$,where the *α*(*E*) is the absorption coefficient, *α*_0_ and *E*_0_ are two constants, and *E* is the photon energy.

**Table S6.** Detailed voltage loss parameters of the binary and ternary OSCs.

| OSCs | *E*_g_  (eV)^a^ | *q*Δ*V*_loss_ (eV) | *qV*_OC, SQ_  (eV) | *qV*_OC, rad_  (eV) | *q*Δ*V*_1_  (eV) | *q*Δ*V*_2_  (eV) | *q*Δ*V*_3_  (eV) | EQE_EL_  (%) | *q*Δ*V*_3_  (eV)^b^ |
| --- | --- | --- | --- | --- | --- | --- | --- | --- | --- |
| PBDB-TCl:AITC | 1.78 | 0.69 | 1.49 | 1.41 | 0.29 | 0.08 | 0.32 | 0.10×10^-2^ | 0.30 |
| PBDB-TCl:AITC: BTP-eC9 | 1.38 | 0.50 | 1.11 | 1.06 | 0.26 | 0.05 | 0.18 | 7.68×10^-2^ | 0.19 |
| PBDB-TCl:BTP-eC9 | 1.38 | 0.52 | 1.12 | 1.06 | 0.26 | 0.06 | 0.20 | 3.25×10^-2^ | 0.21 |

^a^*E*_g_ is the optical bandgap of OSCs calculated on the EQE spectra.

^b^Δ*V*_3_ is calculated from the EQE_EL_.


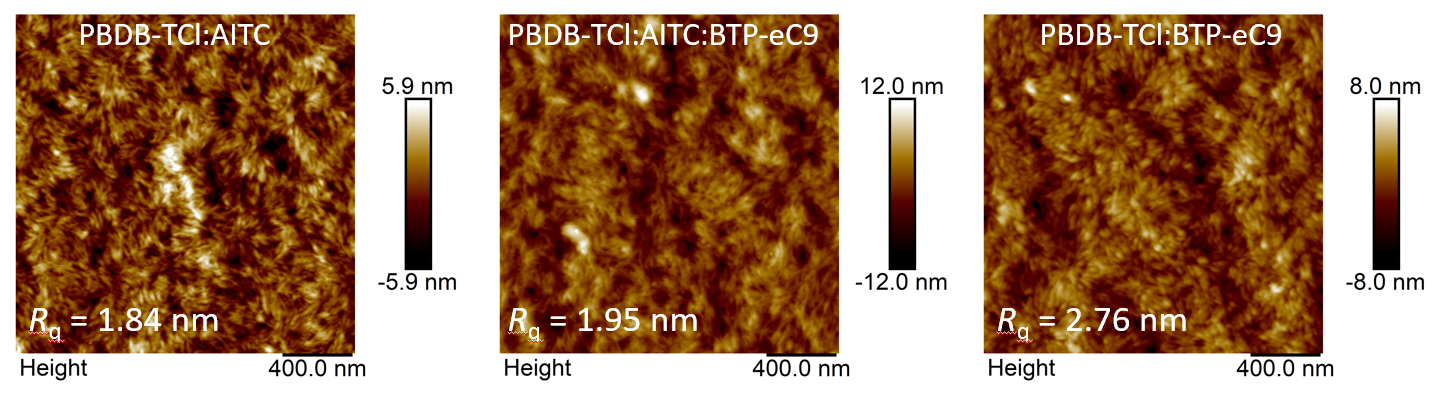


**Fig. S12.** Atomic force microscopy (AFM) height images for three OSCs.


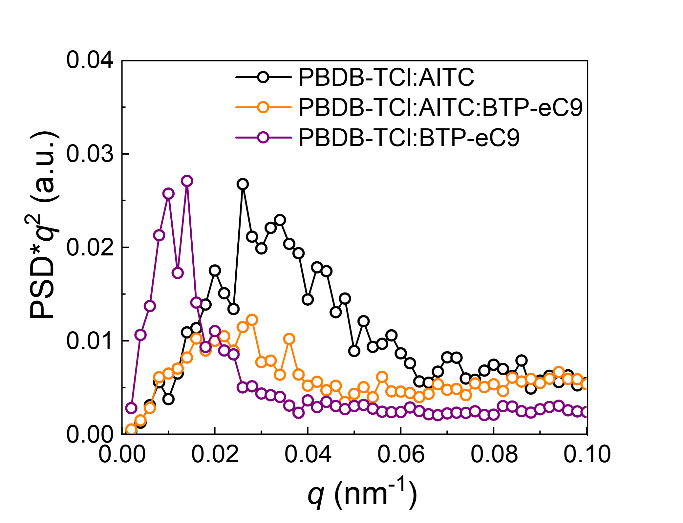


**Fig. S13.** 1D-PSD**q*^2^ spectra of the AFM phase images for the blends, where *q* is spatial frequency.


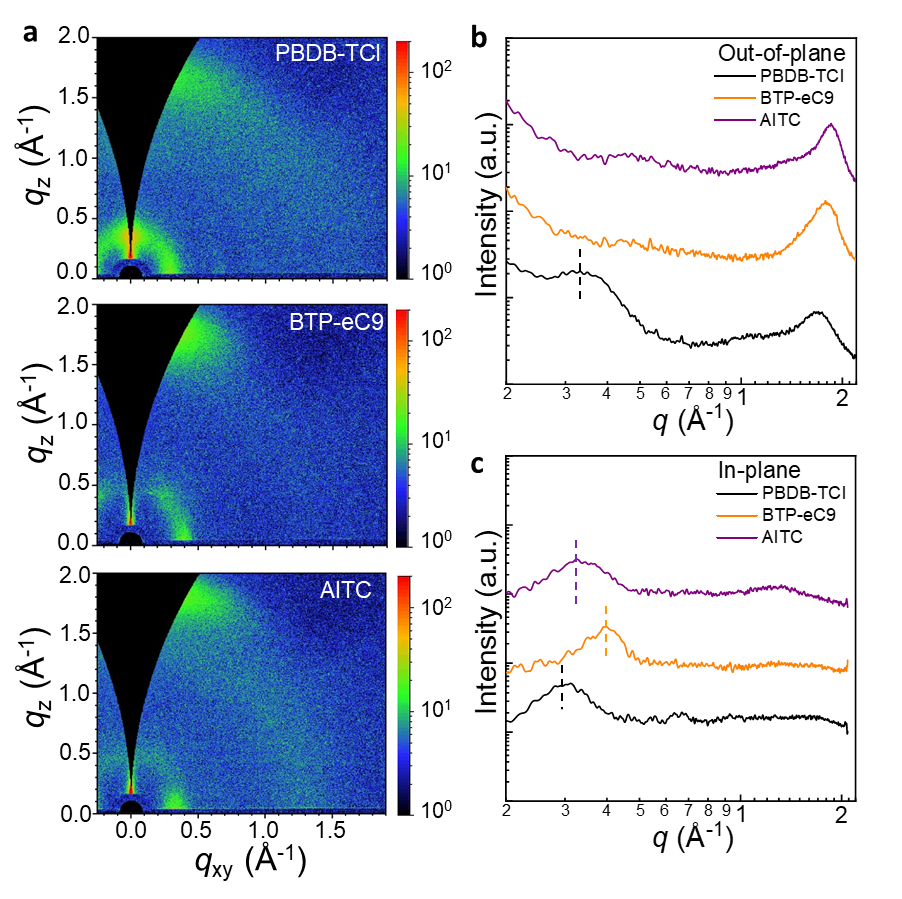


**Fig. S14.** (a) 2D grazing incidence wide angle X-ray scattering (GIWAXS) patterns of the binary and ternary films. (b-c) OOP and IP line-cut profiles of the 2D GIWAXS patterns based on binary and ternary blends.


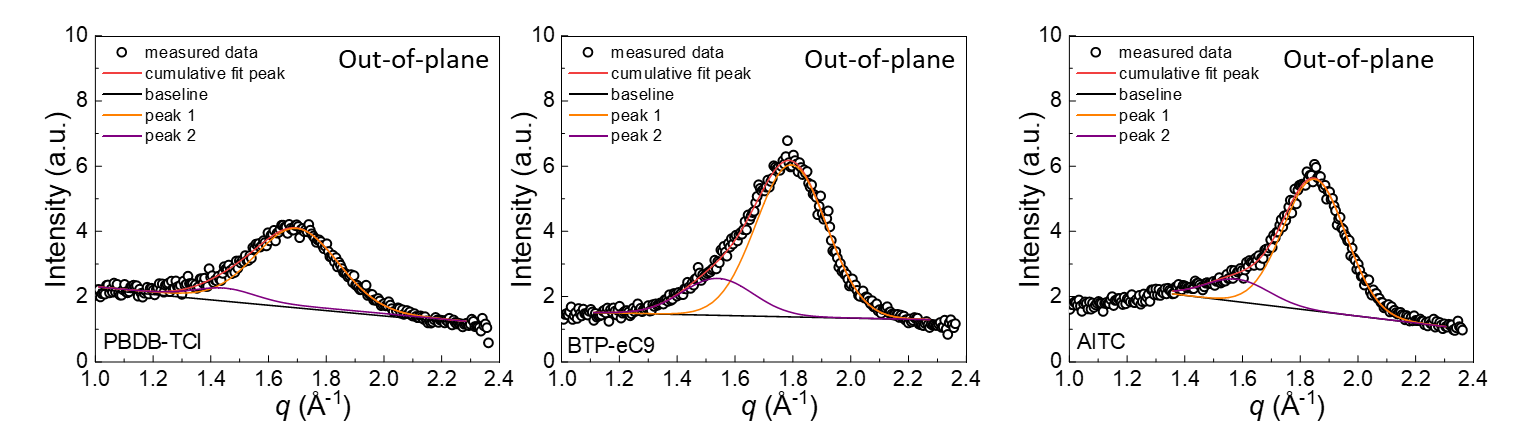
 **Fig. S15.** Line-cut profiles and fitting results in the out-of-plane directions of three BHJ films. The peak 1 refers to π-π stacking peak; peak 2 refers to amorphous phase.

**Table S7.** Detailed GIWAXS peak information of three films.

| films | *q*_Peak1_  (Å^-1^) | *q*_Peak2_  (Å^-1^) | *d*_π-π_  (Å) | FWHM_π-π_  (Å^-1^) | CCL_π-π_  (nm) | area_π-π_ | area_π-π_/h |
| --- | --- | --- | --- | --- | --- | --- | --- |
| PBDB-TCl | 1.698 | 1.450 | 3.700 | 0.336 | 1.683 | 0.897 | 0.897 |
| BTP-eC9 | 1.794 | 1.540 | 3.502 | 0.288 | 1.963 | 1.426 | 1.188 |
| AITC | 1.850 | 1.582 | 3.396 | 0.256 | 2.209 | 1.099 | 1.156 |

The peak 1 refers to π-π stacking peak; peak 2 refers to amorphous phase.


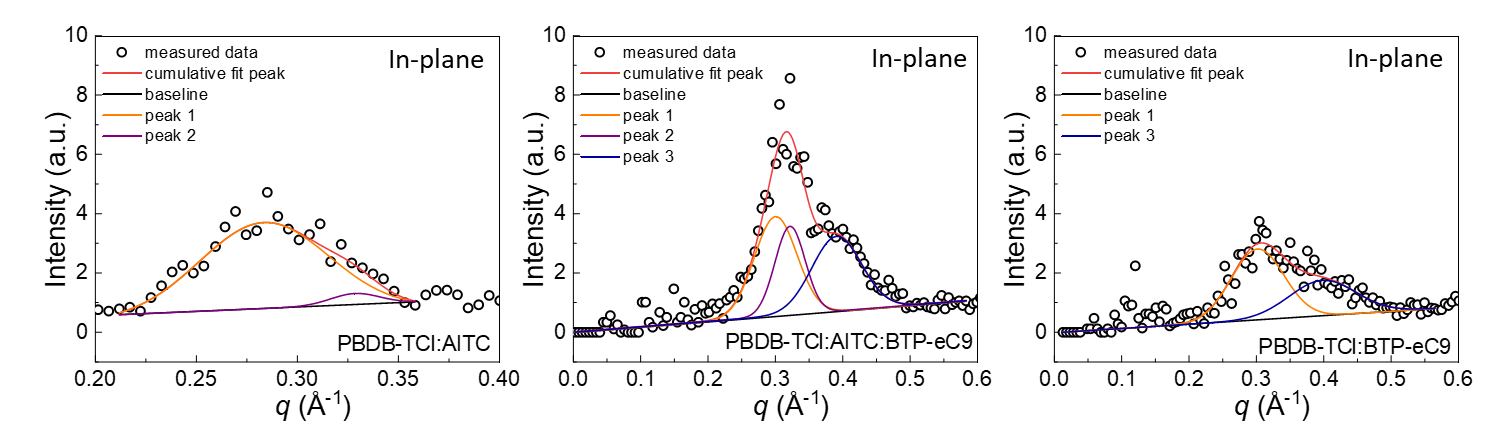


**Fig. S16.** Line-cut profiles and fitting results in the in-plane directions of three BHJ films. The peak 1, 2 and 3 are distributed to PBDB-TCl, AITC and BTP-eC9, respectively.

**Table S8.** Detailed GIWAXS peak information in the in-planes directions of three BHJ films.

| BHJs | *q*_peak1_  (Å^-1^) | *q*_peak2_  (Å^-1^) | *q*_peak3_  (Å^-1^) |
| --- | --- | --- | --- |
| PBDB-TCl:AITC | 0.283 | 0.329 | / |
| PBDB-TCl:AITC:BTP-eC9 | 0.300 | 0.325 | 0.398 |
| PBDB-TCl:BTP-eC9 | 0.302 | / | 0.398 |

The peak 1, 2 and 3 are distributed to PBDB-TCl, AITC and BTP-eC9, respectively.


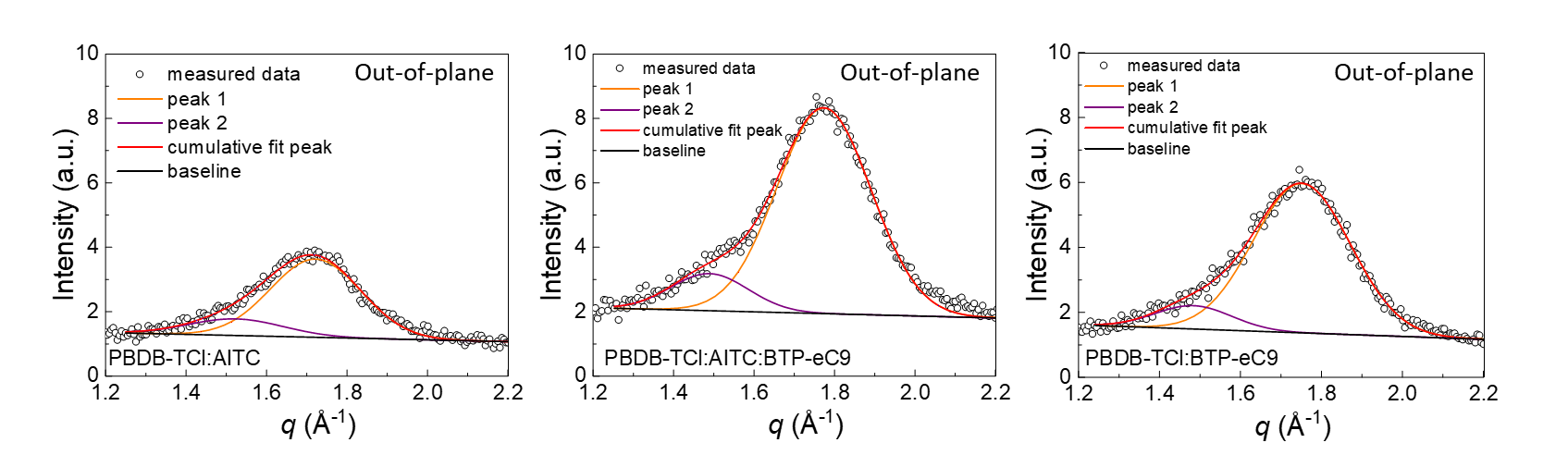


**Fig. S17.** Line-cut profiles and fitting results in the out-of-plane directions of three films. The black and red plots refer to raw data and fitted data, respectively. The baseline of peak near *q* = 1.75 Å^-1^ is established by the linear mode, and the start and terminal of the baseline are the two minimums of the two sides of peak. The peak 1 refers to π-π stacking peak; peak 2 refers to amorphous phase.

**Table S9.** Detailed GIWAXS peak information in the out-of-plane directions of three films.

| Films | *q*_peak 2_  (Å^-1^) | *q*_π-π_  (Å^-1^) | *d*_π-π_  (Å) | FWHM_π-π_  (Å^-1^) | CCL_π-π_  (nm) | Area_π-π_ | Area_peak2_ | Area_π-π_ fraction (%) | Area_π-π_/h |
| --- | --- | --- | --- | --- | --- | --- | --- | --- | --- |
| PBDB-TCl:AITC | 1.52 | 1.72 | 3.65 | 0.26 | 2.38 | 0.68 | 0.15 | 82.0 | 0.58 |
| PBDB-TCl:AITC:BTP-eC9 | 1.49 | 1.77 | 3.54 | 0.28 | 2.28 | 1.88 | 0.28 | 87.2 | 1.58 |
| PBDB-TCl:BTP-eC9 | 1.48 | 1.75 | 3.59 | 0.28 | 2.22 | 1.38 | 0.17 | 89.1 | 1.23 |


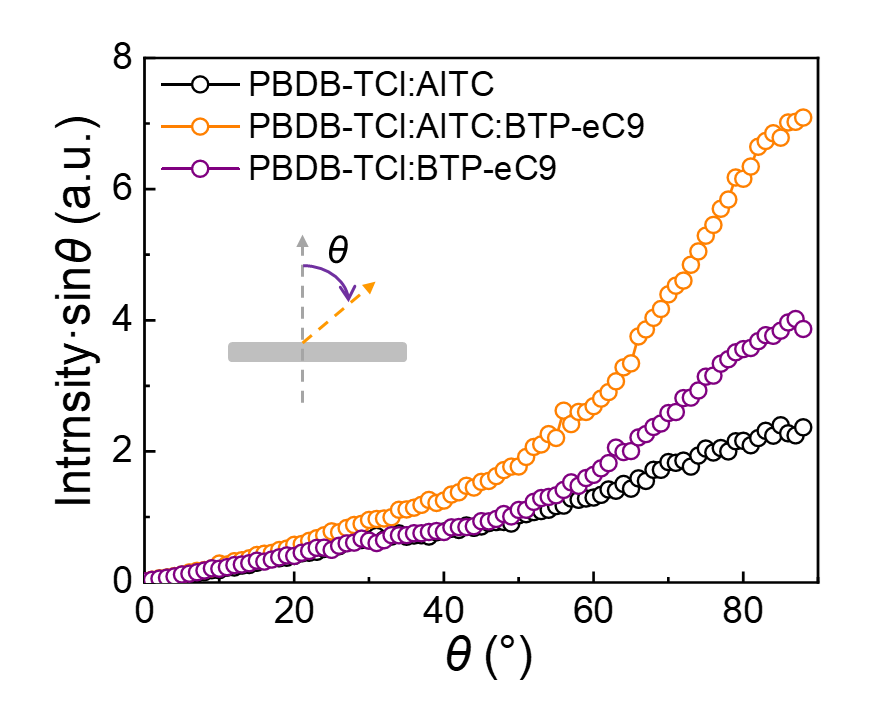


**Fig. S18.** Corrected pole figures extracted from the π-π stacking (010) diffraction peak for the blended films. Definition of polar angle (*θ*) and the ranges corresponding to face-on (A_2_) and edge-on (A_1_) crystallites are shown. *θ* = 0° represents edge-on orientation, and *θ* = 90° represents face-on orientation relative to substrate.


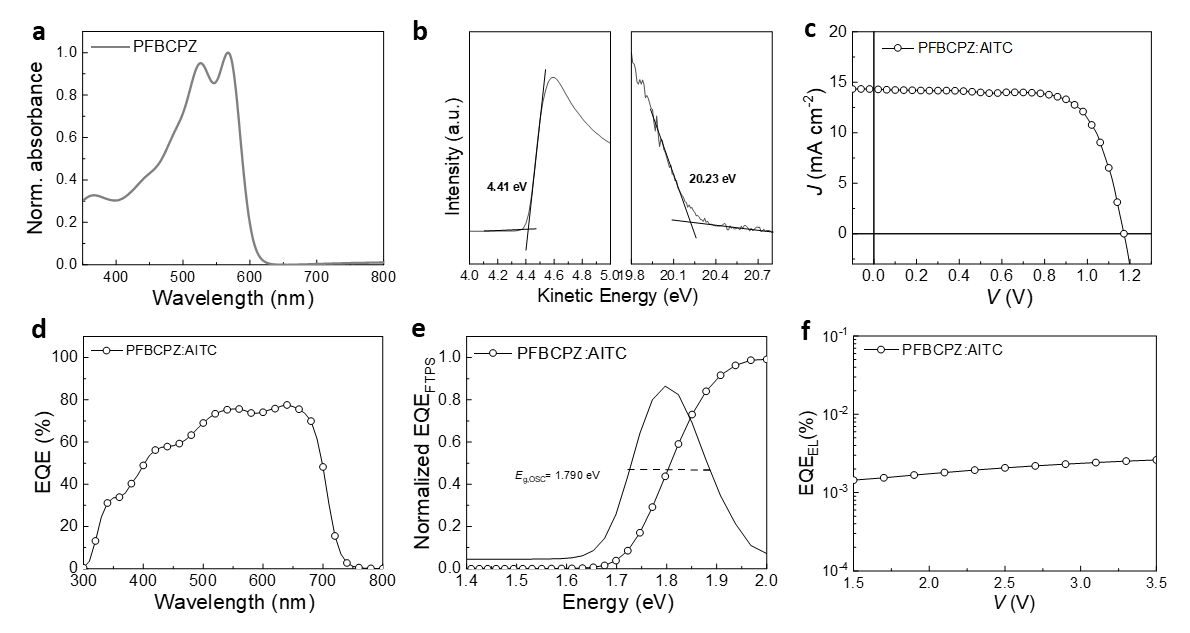


**Fig. S19.** (a) Absorbance spectrum of PFBCPZ film. (b) UPS results of neat PFBCPZ film. (c) *J*-*V* curves, (d) EQE spectra, (e) *E*_g,OSC_ distributions, and (f) EQE_EL_ curve for PFBCPZ:AITC based OSCs.

**Table S10.** Photovoltaic parameters of PFBCPZ:AITC single-junction OSCs.

| OSCs | *V*_OC_ (V) | *J*_SC_/*J*_cal._ (mA cm^-2^)^a^ | FF (%) | PCE (%)^b^ |
| --- | --- | --- | --- | --- |
| PFBCPZ:AITC | 1.17 | 14.3/14.3 | 71.7 | 12.0 (11.6±0.3) |

**Table S11.** Detailed voltage loss parameters of the PFBCPZ:AITC based single-junction OSCs.

| OSCs | *E*_g_  (eV) | *q*Δ*V*_loss_  (eV) | *qV*_OC,SQ_  (eV) | *qV*_OC,rad_  (eV) | *q*Δ*V*_1_  (eV) | *q*Δ*V*_2_  (eV) | *q*Δ*V*_3_  (eV) | EQE_EL_  (%) | *q*Δ*V*_3_  (eV) |
| --- | --- | --- | --- | --- | --- | --- | --- | --- | --- |
| PFBCPZ:AITC | 1.79 | 0.62 | 1.50 | 1.44 | 0.29 | 0.06 | 0.27 | 3.1×10^-3^ | 0.27 |


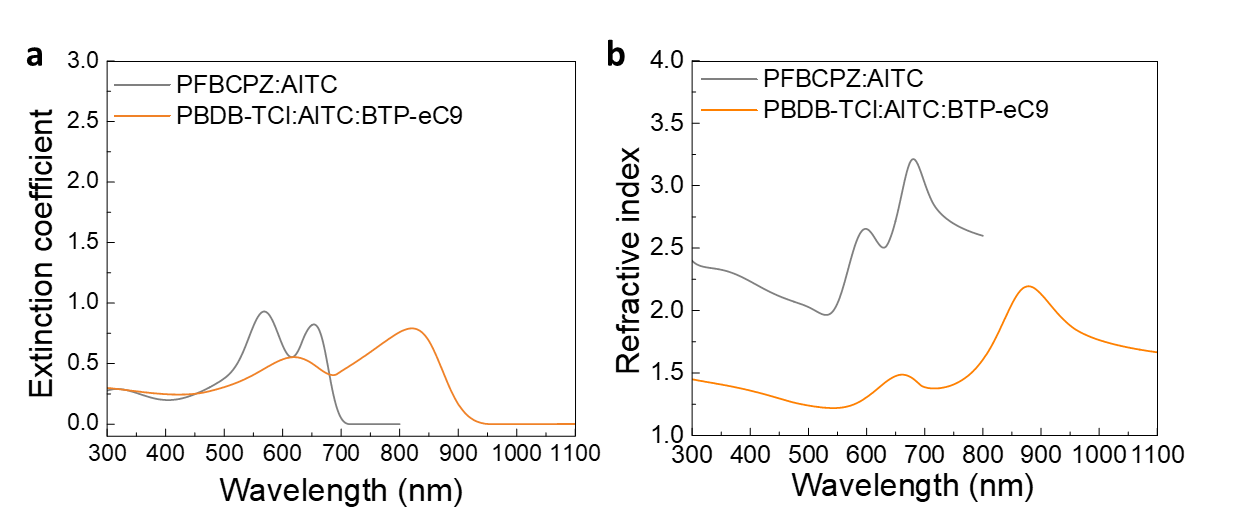


**Fig. S20.** (a-b) The extinction coefficient (*k*) and refractive index (*n*) values of the active layers in tandem solar cells.


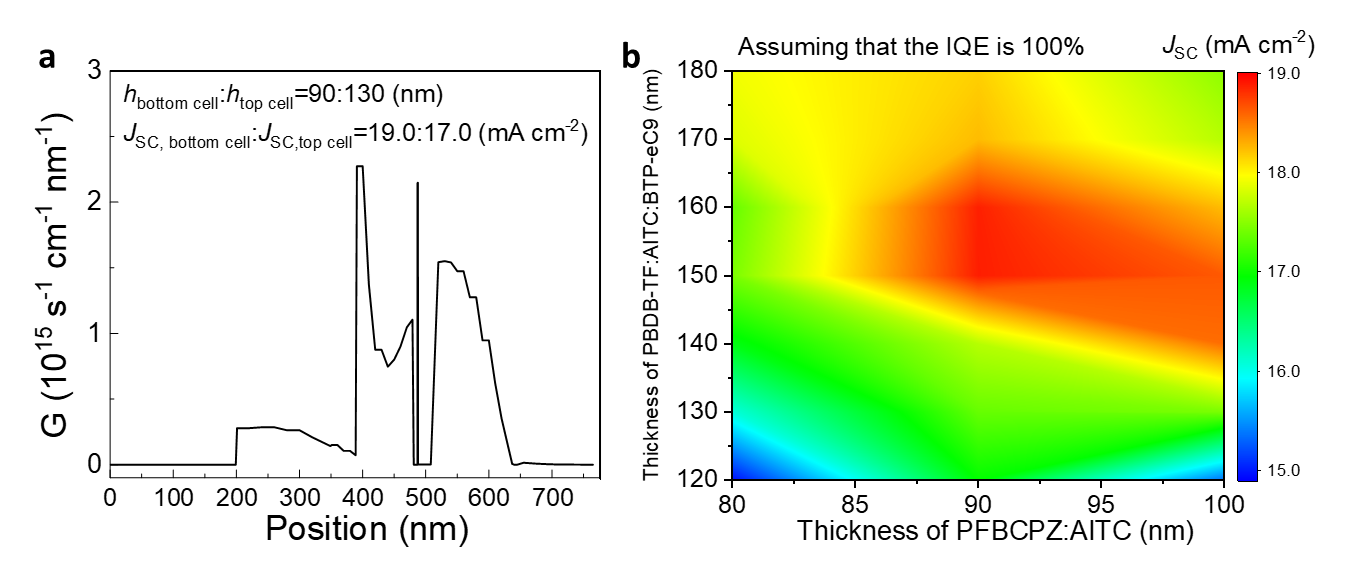


**Fig. S21.** (a) The simulated photon absorption rate (G) in each position of tandem OSCs with sub-cell active layer thicknesses of 90 and 130 nm. (b) Simulated theoretical *J*_SC_ of tandem solar cell as a function of active layer thicknesses of the bottom and top sub-cells.


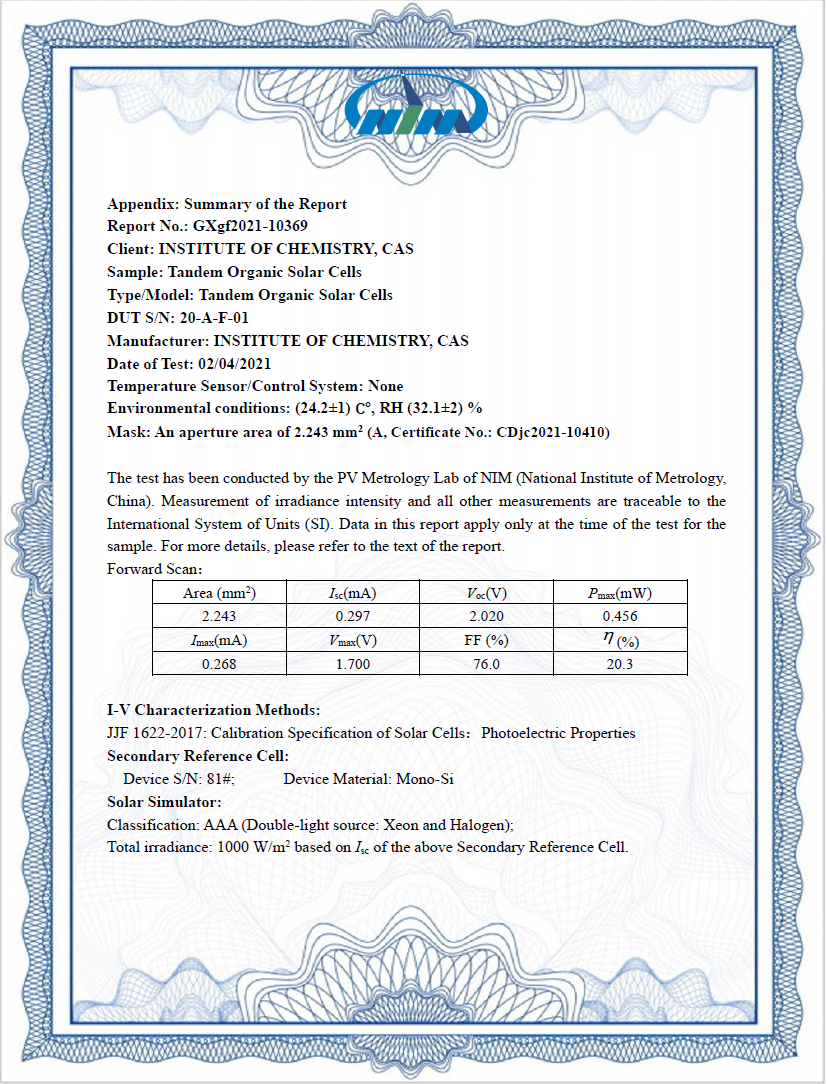


**Fig. S22.** The certification report of the best performance tandem cell processed from NIM, China.

**Table S12.** Photovoltaic parameters of PBDB-TCl:AITC:BTP-eC9 based OSCs under various light intensity.

| Intensity (mW cm^-2^) | *V*_OC_ (V) | *J*_SC_ (mA cm^-2^) | FF (%) | PCE (%) |
| --- | --- | --- | --- | --- |
| 100 | 2.02 | 13.4 | 74.4 | 20.1 |
| 90 | 1.99 | 11.8 | 74.9 | 19.6 |
| 80 | 1.97 | 10.5 | 75.0 | 19.5 |
| 70 | 1.94 | 9.0 | 75.2 | 18.8 |
| 60 | 1.92 | 7.8 | 74.9 | 18.8 |
| 50 | 1.90 | 6.5 | 75.3 | 18.6 |
| 40 | 1.87 | 5.1 | 74.3 | 17.8 |
| 30 | 1.82 | 3.8 | 74.4 | 17.1 |
| 20 | 1.76 | 2.5 | 73.9 | 16.4 |
| 10 | 1.67 | 1.4 | 72.1 | 17.8 |

**REFERENCES**

1. Chen Z. [Wang](https://www.researchgate.net/profile/Tong-Wang-68?_sg%5B0%5D=BT_dmGx6YCoudaVwKAw5rIGdt0NmjnQJ7E_MdnPLkbzjNZfRXKvkugDhu_yFPUYkKcae-IQ.Z8zIJQn1ajo5HW--2be3n-u3kDxQxg3mlzSeZgo9du7eXaevhBAAd2XHbGDXhC4oSYjOTX8b1_MwWAFzID77HQ&_sg%5B1%5D=fqUhSl2OxbefGCPvuiJlyDrceiSetVNvp7rVTTXsqvkG5mb294pCAg33dKd8ybepKBQf_Kk.hJZu2kePXycAOmO-YL7IafeN8gH_xvORljcGky9-cMzbFml50giedjxc1jugPqrBzCxMecCw3wJkIJwHvKyogA) T and [Wen](https://www.researchgate.net/profile/Zhenchuan-Wen?_sg%5B0%5D=BT_dmGx6YCoudaVwKAw5rIGdt0NmjnQJ7E_MdnPLkbzjNZfRXKvkugDhu_yFPUYkKcae-IQ.Z8zIJQn1ajo5HW--2be3n-u3kDxQxg3mlzSeZgo9du7eXaevhBAAd2XHbGDXhC4oSYjOTX8b1_MwWAFzID77HQ&_sg%5B1%5D=fqUhSl2OxbefGCPvuiJlyDrceiSetVNvp7rVTTXsqvkG5mb294pCAg33dKd8ybepKBQf_Kk.hJZu2kePXycAOmO-YL7IafeN8gH_xvORljcGky9-cMzbFml50giedjxc1jugPqrBzCxMecCw3wJkIJwHvKyogA) Z *et al.* Trap state induced recombination effects on indoor organic photovoltaic cells. *ACS Energy Lett.* 2021; **6**: 3203-11.
